# Genome-Wide Prediction of SH2 Domain Targets Using Structural Information and the FoldX Algorithm

**DOI:** 10.1371/journal.pcbi.1000052

**Published:** 2008-04-04

**Authors:** Ignacio E. Sánchez, Pedro Beltrao, Francois Stricher, Joost Schymkowitz, Jesper Ferkinghoff-Borg, Frederic Rousseau, Luis Serrano

**Affiliations:** 1 European Molecular Biology Laboratory, Heidelberg, Germany; 2 EMBL-CRG Systems Biology Unit, CRG-Centre de Regulacio Genomica, Barcelona, Spain; 3 Switch Laboratory, Flanders Interuniversity Institute for Biotechnology (VIB), Brussels, Belgium; 4 Nordita, Copenhagen, Denmark; Columbia University NY, United States of America

## Abstract

Current experiments likely cover only a fraction of all protein-protein interactions. Here, we developed a method to predict SH2-mediated protein-protein interactions using the structure of SH2-phosphopeptide complexes and the FoldX algorithm. We show that our approach performs similarly to experimentally derived consensus sequences and substitution matrices at predicting known *in vitro* and *in vivo* targets of SH2 domains. We use our method to provide a set of high-confidence interactions for human SH2 domains with known structure filtered on secondary structure and phosphorylation state. We validated the predictions using literature-derived SH2 interactions and a probabilistic score obtained from a naive Bayes integration of information on coexpression, conservation of the interaction in other species, shared interaction partners, and functions. We show how our predictions lead to a new hypothesis for the role of SH2 domains in signaling.

## Author Summary

Understanding the functional role of every protein in the cell is a long-standing goal of cellular biology. An important step in this direction is to discover how and when proteins interact inside the cell to accomplish their tasks. Many of the cellular functions depend on reversible protein modifications like phosphorylation. To sense these modifications, cells have protein domains capable of binding phosphorylated proteins such as the SH2 domain. In this work, we show that it is possible to use the three-dimensional structure of protein domains to predict its binding preferences. Using a computational tool called FoldX, we have predicted the binding specificity of several human SH2 domains. These predictions, based on the computational analysis of the 3-D structure, were shown to be of similar accuracy as those obtained from experimental binding assays. We show here that it is also possible to understand how a mutation changes the binding preference of protein binding domains, opening the way for better understanding of some disease causing mutations. The combination of this novel computational approach with other sources of information allowed us to provide a set of high-confidence novel interactions for the proteins here studied.

## Introduction

The cell's ability to respond to internal and external cues depends largely on reversible post-translational modifications of proteins, such as phosphorylation, ubiquitylation, methylation or acetylation. These modifications often occur on short unstructured stretches of proteins and are read by domains that recognize the modified form [1]. Signal transduction often involves phosphorylation of tyrosine residues by tyrosine kinases. This turns on the recognition of the phosphorylated site by SH2-domain containing proteins, leading to regulation of cellular localization, enzymatic activity and formation of multiprotein complexes [2],[3].

Experiments using peptide libraries indicate that each SH2 domain binds a different spectrum of phosphopeptides [4]–[8]. Although the differences in the binding constants for different phosphopeptides are often modest [9], they are known to play an important role in regulating signal transduction *in vivo* [3]. For example, exchanging an SH2 domain for another with a different specificity can impair activation of the Ras pathway in *Caenorhabditis elegans* [10], alter the transformation ability of the Abelson murine leukemia [11] and the Rous sarcoma viruses [12] and trigger mesoderm formation in *Xenopus laevis* [13]. Moreover, point mutations that induce changes in specificity are associated with diseases such as the X-linked alpha-gammaglobulinemia [14], the X-linked lymphoproliferative syndrome [15] and the Noonan syndrome [16].

The in vitro binding specificity of SH2 domains is commonly determined using peptide libraries [4],[17]. The results of peptide library experiments are often summarized in the form of consensus sequences [4] or as position-specific scoring matrices [18] and then used to predict and characterize novel *in vivo* SH2-mediated protein-protein interactions. However, the genome-wide determination of the binding specificity of SH2 domains using peptide libraries seems impractical given the more than one hundred human SH2 domains [19] and the limited complexity of the peptide libraries available. The computational modeling of SH2 domain specificity is in a developing stage [20]–[22]. On one hand, fast methods with energy functions based on solvent-accessible surface area reached only limited success [20]. On the other hand, algorithms using molecular dynamics [21] and comparative molecular field analysis [22] showed a good predictive power but are computationally expensive and can only be used to study a limited number of complexes for a given SH2 domain. Recently, McLaughlin and coworkers predicted the binding specificity of two SH2 domains by combining information on known binding peptides with structure-based calculations [23]. The resulting hidden Markov models could be used in a genomic scale to predict SH2-mediated interactions [23]. However, a main drawback of their method is that it relies partially on experimental information. The limitations of the current computational methods encouraged us to develop a new structure-based algorithm to predict the specificity of SH2 domains.

Our group has developed FoldX, an empirical force field for the prediction of protein energetics [24]. The energy of a protein or protein complex is calculated in FoldX using a structure-based energy function. This energy function is a linear combination of empirical terms such as solvation of polar and hydrophobic atoms, water binding, Van der Waals energy, steric clashes, hydrogen bonds, electrostatic interactions and side chain and main chain entropy. These energy terms are scaled with atom or residue burial and have empirical weights derived by fitting to a database with more than one thousand mutations [24]. FoldX can give accurate predictions for changes in protein stability upon mutation [24], water and metal binding [25] and interactions between globular domains [26]–[29]. The algorithm is fast enough to be used in genome-wide predictions and the modularity of its energy function makes the implementation of new capabilities straightforward. FoldX is available online at http://foldx.crg.es.

We have implemented the force field contributions of phosphorylated amino acids (pTyr, pSer and pThr) into FoldX and used it to predict the binding specificity of nine human SH2 domains with known structure. Our calculations can reproduce experimental consensus target sequences. FoldX performs as well as experimentally derived consensus sequences or position-specific substitution matrices in the prediction of *in vitro* SH2-phosphopeptide binding and *in vivo* SH2-mediated protein-protein interactions. Together with information on phosphorylation and secondary structure, FoldX can give accurate predictions of novel protein-protein interactions. We used the developed method to predict a high confidence SH2 interaction network and validated it using information on co-expression, conservation of the interaction in other species, shared interaction partners and shared GO functions, integrated using a naive Bayes network. The predicted interactions can be use to derive biologically relevant testable hypothesis.

## Results

### Implementation of Phosphorylated Residues into FoldX

We have implemented phosphorylation of tyrosine, serine and threonine residues into FoldX [24] by combining available experimental information and empirical estimates (see Methods). We have validated our implementation in two ways. First, we predicted the change in the free energy of binding upon dephosphorylation for nineteen protein-phosphopeptide complexes [30]–[41] (Table S1). Experimentally, nine of the complexes do not form at all, or are severely destabilized (>5 kcal/mol) if the peptide is not phosphorylated. The average predicted change in free energy for these complexes is 6.8±2.5 kcal/mol (average±stdev). For the other ten complexes, the average experimental change in the free energy of binding is 0.97±0.61 kcal/mol (average±stdev). The average predicted change in free energy for these complexes is 1.7±1.5 kcal/mol (average±stdev). Thus, FoldX can predict whether a protein-phosphopeptide complex will be disrupted or not by dephosphorylation.

**Figure 1:**
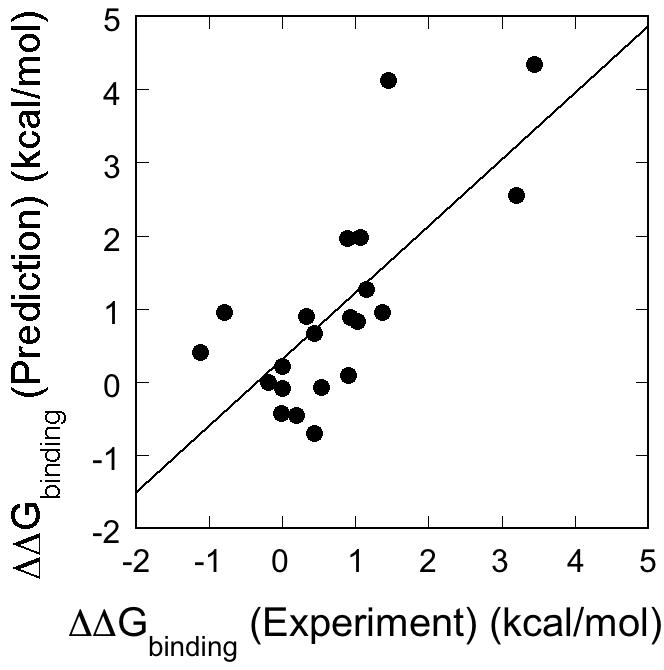
Prediction of the changes in free energy for the formation of protein-phosphopeptide complexes upon mutation of protein residues in the environment of the phosphate group. The fitted line has a correlation R-value of 0.72 and a slope of 0.91.

Second, we have predicted the changes in the free energy of formation of 21 protein-phosphopeptide complexes upon mutation of protein residues close to a phosphorylated residue [15], [34], [42]–[45] (Table S2). The experimental changes in the free energy of binding range from −1.13 to 3.44 kcal/mol. Figure 1 shows the correlation between the experimental and calculated changes in free energy of binding upon mutation. A linear fit of the data gives a correlation R-value of 0.72, a slope of 0.91 and a standard deviation of 0.95 kcal/mol. The quality of the predictions is comparable to that of changes in protein stability upon mutation [24], confirming that FoldX can be used to predict the energetics of phosphorylated residues.

### FoldX Predictions Reproduce Experimental Consensus Target Sequences

Next, we tested the ability of FoldX to predict the binding specificity of phosphopeptide-binding domains. The binding specificity of a domain is commonly determined *in vitro* by exposing the domain to a synthetic phosphopeptide library in which several positions have been randomized. The preferred residues at each position and the consensus target sequence are identified by sequencing the pool of bound peptides [4]. We considered here the nine human SH2-phosphopeptide complexes of known three-dimensional structure (Table 1), for which eight experimental consensus sequence patterns are available [4]-[8] (Table 1). All eight consensus peptides bind the corresponding SH2 domain [4]–[8], which strongly suggests that most sequences matching a consensus will bind the domain. On the other hand, the comparison of the experimental consensus sequences and the crystallized sequences (Table 1) clearly shows that there are sequences that do not match the consensus and yet bind the target SH2 domain. This is in agreement with the heterogeneous pool of bound peptides found in library experiments with SH2 domains [4]–[8].

**Table 1:** Comparison of experimental consensus target sequences with FoldX predictions of binding specificity for human SH2 domains.

SH2 domain	Structure	Crystallized Sequence	Consensus Target Sequence	ΔG_binding_ (Consensus)	ΔG_binding_ (Random)	*p* (Random Better than Consensus)
p85	2IUH	TNEpYMDMK	pY[MLI]XM [4]	2.24±1.39	9.74±3.25	0.01
Lck	1CWE	QpYEEIP	pYEEI [5]	1.13±0.90	6.76±2.61	0.01
Src	1SPS	PQpYEEIP	pYE[ENY][IML] [4]	2.62±1.47	7.33±2.67	0.02
Grb2	1ZFP	EpYINQ	pY[QY]NY [5]	1.06±0.71	7.50±2.87	0.02
Sap	1D4W	SLTIpYAQVQK	TXpYXX[IV] [6]	5.63±2.80	12.25±5.45	0.08
Syk (C-term)	1A81	PDpYEPIRKGQRD	pY[QTE][QTE]L [8]	4.19±2.15	6.97±2.29	0.11
Nck1	1CI9	HIpYDEVAAD	pYDE[PDV] [4]	2.31±2.33	5.14±2.57	0.14
Stat1	1YVL	pYDKPH	pYERQH [7]	1.10	1.14±1.71	0.48
Syk (N-term)	1A81	DLpYSGLN	—	—	1.95±1.87	—

Binding energies are relative to the crystallized peptide and in kcal/mol units.

We have used position specific scoring matrices calculated with FoldX to compute the binding energy of 50,000 random sequences and 50,000 sequences matching the experimental consensus (see Methods). The average binding free energies for both classes of peptides are shown in Table 1. In all cases, peptides matching the consensus pattern are predicted to bind better than peptides of random sequence. A variable fraction of random peptides is predicted to bind better than the average of peptides matching the consensus (Table 1). These predictions may be due to the consensus target sequences not covering all possible binding sequences, to the crystallized sequence being a bad template for sequences matching the consensus or to modeling errors. Overall, the predictions from FoldX are in agreement with the experimental binding specificity of these eight SH2 domains.

### FoldX Prediction of *in vitro* SH2 Domain-Phosphopeptide Interactions

**Figure 2:**
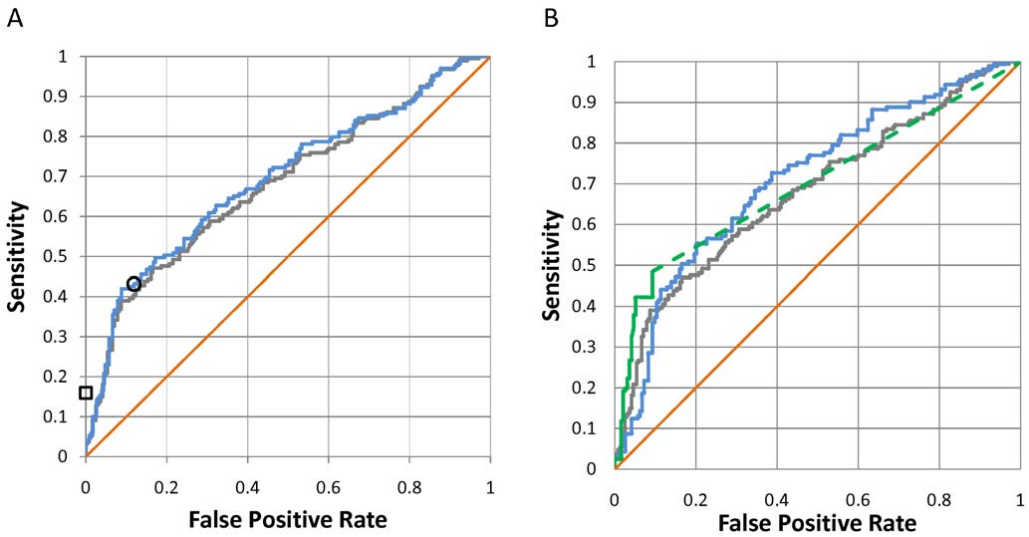
Prediction of SH2 domain-phosphopeptide interactions using FoldX and experimental data from peptide libraries. The orange line corresponds to random prediction, the grey line to prediction for all nine domains in Table 1. (A) Comparison of predictions from experimental consensus sequences allowing zero (square) and one mismatch (circle) with the consensus sequence with FoldX predictions for the eight domains in Table 1 for which a consensus sequence is available (blue line). (B) Comparison of predictions from Scansite scoring matrices for the Nck1, p85, Src, Lck, and Grb2 SH2 domains (green line) with FoldX predictions for the same domains (blue line).

We have made a direct comparison between experimental SH2 domain binding specificity and FoldX predictions using experimental binding affinities of SH2 domains for non-randomized peptides. We have retrieved a list of 429 phosphopeptides tested for binding to the nine SH2 domains in Table 1 from the ADAN database (http://adan.embl.de, Table S3). 187 of the protein-phosphopeptide complexes have a measurable affinity under the conditions tested and were taken as the positive dataset. The other 242 complexes do not form under the conditions tested and were taken as the negative dataset. We have computed the binding energy of all putative complexes using position specific scoring matrices calculated with FoldX, relative to the average binding energy of 50,000 random peptides. We generated a ROC curve by considering as positives peptides with different relative binding energies (grey line in Figure 2). The area under the ROC curve for the FoldX predictions is 0.68±0.03 (statistics obtained using the SPSS package under the nonparametric assumption and a confidence level of 95%, results for the individual domains are shown in Table S4). The probability of the true area being 0.5 (random prediction) is 1.3⋅10^−10^, indicating that FoldX can predict *in vitro* binding of phosphopeptides to SH2 domains.

We have made a direct comparison of FoldX and experimental consensus target sequences in the detection of protein-phosphopeptide complexes for the eight domains in Table 1 for which a consensus sequence is available (Figure 2A). Predictions using experimental target sequences allowed zero (square) and one mismatch (circle) with the consensus sequence. The performance of FoldX (blue line) over the set of 169 positives and 227 negatives is similar to that of experimental consensus sequences, with an area under the ROC curve of 0.70±0.03 (p-value 3.1⋅10^−11^). Experiments with randomized peptide libraries can also be used to generate position-specific scoring matrices [18]. Figure 2B compares the predictions from FoldX (blue line) with the predictions from Scansite scoring matrices [18] for five of the domains in Table 1 (green line, Table S4). This dataset includes 131 positives and 164 negatives for the Nck1, p85, Src, Lck and Grb2 SH2 domains. The area under the ROC curve is 0.71±0.03 (p-value 6.2⋅10^−12^) for the FoldX predictions and 0.70±0.03 (p-value 8.6⋅10^−11^) for the predictions using experimental scoring matrices. Altogether, the performance of our structure-based calculations in the prediction of *in vitro* protein-phosphopeptide binding specificity is similar to experimental methods based on peptide libraries.

### FoldX Prediction of Changes in Specificity in the Src SH2 Domain Upon Mutation

The binding specificity of the Src SH2 domain changes from pYEEI-containing phosphopeptides to pYVNV-containing phosphopeptides upon mutation of threonine EF1 to tryptophan [46]. We have used the structure of the mutated Src SH2 domain in complex with a pYVNV-containing phosphopeptide (1F1W.pdb) to further test the ability of FoldX to predict the binding specificity of SH2 domains. We calculated a position-specific substitution matrix for the ThrEF1Trp Src SH2 domain using FoldX and compared it to the substitution matrices for the wild type Src and Grb2 SH2 domains in two ways. First, we calculated the binding energy for the complexes of the three domains with all tyrosine-containing peptides in the human genome. The binding energies for the ThrEF1Trp Src SH2 domain show a strong correlation with the Grb2 SH2 domain and a weak one with the Src SH2 domain (Table 2). Thus, FoldX predicts that the binding specificity of the ThrEF1Trp Src SH2 domain is Grb2-like, as observed experimentally [46]. Second, we tested the ability of the substitution matrix for the ThrEF1Trp Src SH2 domain to discriminate between peptides positive and negative for binding to the Src and Grb2 SH2 domains (Table 2). The area under the ROC curve for prediction of binding to the Grb2 domain is 0.69, much higher than for the wild type Src SH2 matrix (AROC 0.29) and close to the Grb2 SH2 matrix (AROC 0.82). At the same time, the matrix for the ThrEF1Trp Src SH2 domain is a bad predictor for binding to the Src domain (AROC 0.35), clearly worse than the wild type matrix (AROC 0.64). We conclude that FoldX can predict the change in *in vitro* binding specificity induced by the ThrEF1Trp mutation in the Src SH2 domain.

**Table 2:** FoldX prediction of the change in specificity of the Src SH2 domain ThrEF1Trp mutant.

SH2 domain	Correlation R-Value for the Predicted Binding Free Energies to all Tyrosines in the Human Genome			Area Under the ROC Curve for Prediction of In Vitro SH2-Phosphopeptide Binding	
	Src	ThrEF1Trp Src	Grb2	Grb2 peptides	Src peptides
Src	1	0.26	0.18	0.29	0.64
ThrEF1Trp Src	0.26	1	0.76	0.69	0.35
Grb2	0.18	0.76	1	0.82	0.54

### FoldX Prediction of *in vivo* SH2-Mediated Protein-Protein Interactions

**Figure 3:**
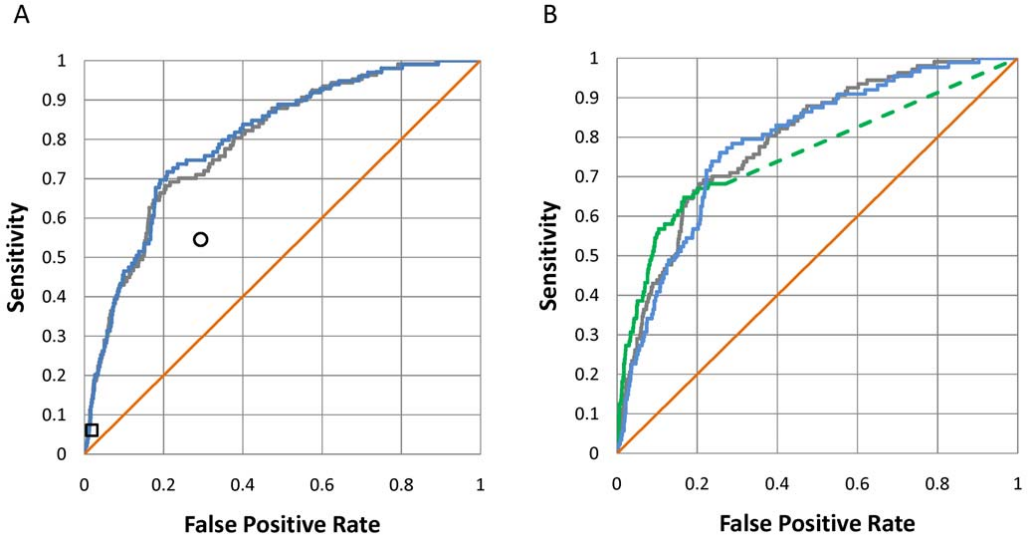
Prediction of SH2-mediated protein-protein interactions using FoldX and experimental data from peptide libraries. The orange line corresponds to random prediction, and the grey line to prediction for all nine domains in Table 1. (A) Comparison of predictions from experimental consensus sequences allowing zero (square) and one mismatch (circle) with the consensus sequence with FoldX predictions for the eight domains in Table 1 for which a consensus sequence is available (blue line). (B) Comparison of predictions from Scansite scoring matrices for the Nck1, p85, Src, Lck, and Grb2 SH2 domains (green line) with FoldX predictions for the same domains (blue line).

We showed so far that FoldX can predict the binding *in vitro* of phosphopeptides to a given SH2 domain for which high resolution structural data is available. Next, we used FoldX for the prediction of binding *in vivo*. We compiled a list of SH2-mediated protein-protein interactions in the following way: First, we extracted from the Human Protein Reference database all interactions for proteins containing the SH2 domains in Table 1. We then curated the database to keep only interactions known to be mediated by the SH2 domains. The final list of positives contains 107 interactions for the nine proteins (see Table S5). All human proteins not reported as positives were taken as negatives. We have used FoldX matrices to compute the binding energy of each of the 499,293 putative complexes (55,477 tyrosines in the human genomes times 9 SH2 domains), relative to the average binding energy of 50,000 random peptides. The predicted binding energy of an SH2 domain with a putative target protein was considered to be the same as the most favorable binding peptide within that protein. We generated a ROC curve by considering as positives target proteins with different relative binding energies (grey line in Figure 3; results for the individual domains shown in Table S4). The area under the ROC curve for the FoldX predictions is 0.79±0.02, (p-value 9.2⋅10^−26^), indicating that FoldX is able to predict *in vivo* SH2-mediated protein-protein interactions.

We have compared the ability of FoldX, experimental consensus target sequences and Scansite matrices to identify *in vivo* SH2-mediated protein-protein interactions (Figure 3). There are 99 positives for the eight domains in Table 1 for which a consensus sequence is available. Predictions using experimental target sequences were made allowing zero (Figure 3A, square) and one mismatch (Figure 3A, circle) with the consensus sequence. The performance of FoldX (Figure 3A, blue line) is similar to that of experimental consensus sequences, with an area under the ROC curve of 0.80±0.02 (p-value 4.5⋅10^−25^). The results for the five domains with available Scansite matrices (88 positives) are shown in Figure 3B and Table S4. The area under the ROC curve is 0.79±0.02 (p-value 9.0⋅10^−21^) for FoldX and 0.76±0.03 (p-value 8.3⋅10^−17^) for the Scansite predictions. As observed for *in vitro* interactions, FoldX performs similar to experimental methods based on peptide libraries in the prediction of SH2-mediated *in vivo* interactions.

### Combining FoldX with Information on Phosphorylation State, Secondary Structure, and Conservation

**Figure 4:**
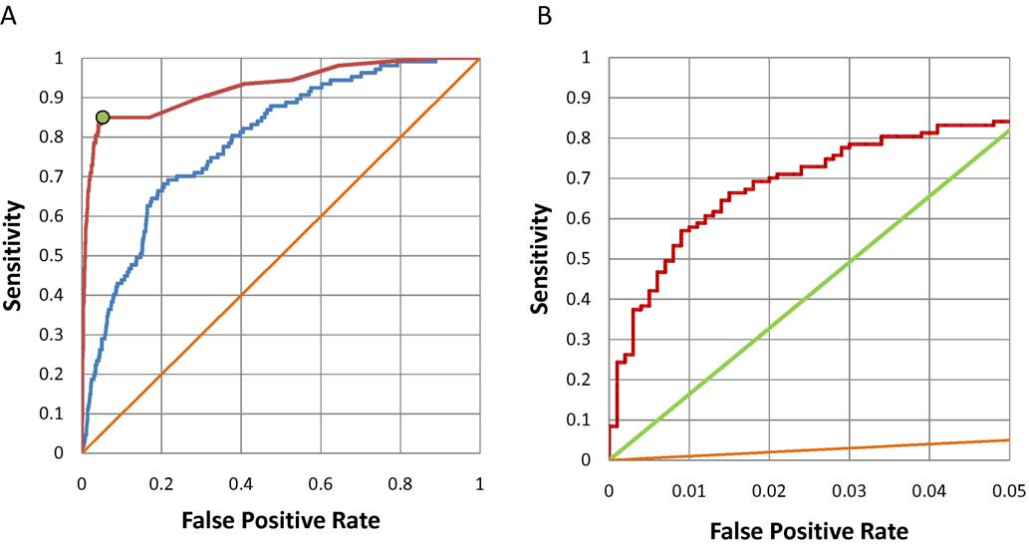
Prediction of SH2-mediated protein-protein interactions using FoldX, phosphorylation, and secondary structure information. (A) FoldX predictions for all nine domains in Table 1 (blue line), predictions using FoldX and the phosphorylation/secondary structure filter (red line), predictions using the phosphorylation/secondary structure filter only (green point), and random prediction (orange line). (B) Detail of the low false positive rate region of (A), with predictions using the phosphorylation/secondary structure filter (green curve) and both the filter and FoldX (red curve).

The final goal of our work is to make useful predictions of SH2-mediated protein-protein interactions. SH2 target sites are likely to be not only phosphorylated, but also within disordered regions of proteins [47]. In order to increase the accuracy of the predictions from FoldX, we filtered our predictions for sites known to be phosphorylated [48]–[50] or predicted by the disphos algorithm [47] to be phosphorylated and within a disordered region. The results are shown in Figure 4A. The area under the ROC curve for prediction of SH2 target proteins increases from 0.79±0.02, (p-value 9.2⋅10^−26^) for the unfiltered FoldX predictions (blue line) to 0.93±0.02, (p-value 7.9⋅10^−53^) for the filtered predictions (red line). As a control, we tried to predict the same set of interactions using only the phosphorylation/secondary structure filter (Figure 4A, green point). Figure 4B shows a zoom into the low false positive rate region of Figure 4A, with the ROC curve for predictions using the phosphorylation/secondary structure filter (green curve) and using both the filter and FoldX (red curve). FoldX clearly improves the performance of the phosphorylation/secondary structure filter, supporting the combined use of both prediction methods.

Previous work on SH3-mediated interactions suggested that conservation of the prediction in related genomes could be used as an additional empirical filter [51]. We have tested this idea in the case of SH2-mediated interactions using a group of 9 genomes of varying divergence from human (see Methods). The conservation filter improves the predictions from FoldX only slightly and only in the absence of the phosphorylation/secondary structure filter (Figure S1). We suggest that the evolution of SH2 target sites is too fast to give a useful conservation signal in the framework of our method.

### High-Confidence Predictions of SH2-Mediated Protein-Protein Interactions

**Figure 5:**
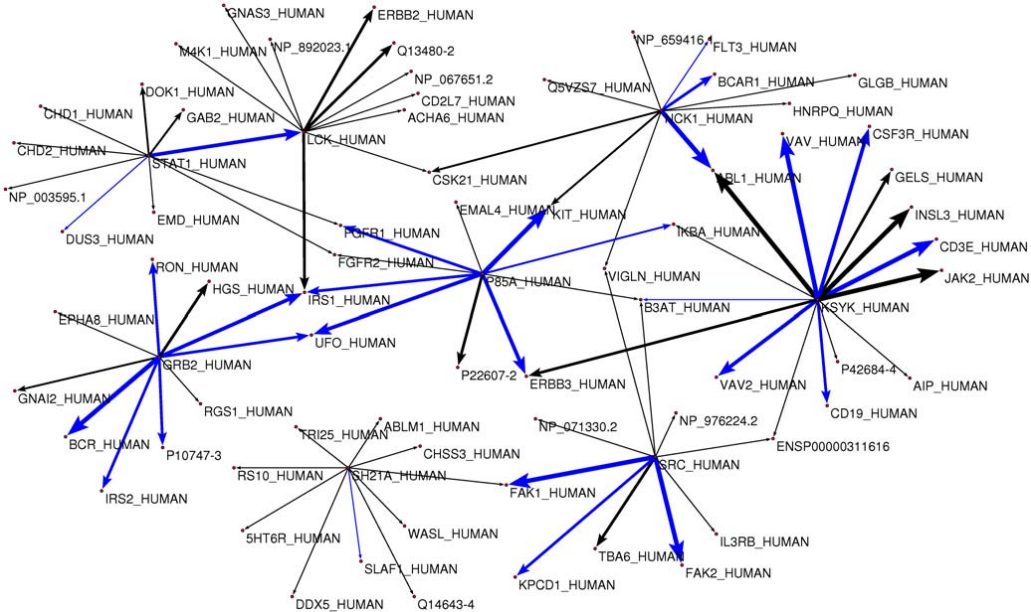
High-confidence predictions of SH2-mediated protein-protein interactions. Arrows depict the predicted interactions from SH2-containing proteins to the top 10 putative targets. The 15 predictions for Syk (KSYK_HUMAN) correspond to the top 10 predictions for both the N-terminal and C-terminal SH2 domains. Both domains were predicted to bind B3AT_HUMAN, ERBB3_HUMAN, VAV_HUMAN, CD19_HUMAN, and IKBA_HUMAN. Interactions confirmed by experimental evidence are highlighted in blue. The thickness of the line is proportional to the odds that the proteins interact *in vivo* as calculated by the naive Bayes network. The exact odds score and the source of the supporting evidence found for each particular interaction is detailed in Table S6. Eight alpha-tubulin subunits were predicted to interact with the Src SH2 domains, and seven guanine nucleotide binding subunits were predicted to interact with the Grb2 SH2 domain. In both cases, these predictions are depicted as a single arrow.

We have obtained a list of highly accurate predicted interactions by running our method with the phosphorylation/secondary structure filter and selecting for each domain the ten targets with the lowest predicted binding energy (Figure 5, Table S6, and Methods). 27 of the 85 predicted interactions (32%) are known physical interactions (for the full proteins) included in the Human Protein Reference Database [49]. We assessed the quality of the predictions by integrating available information regarding co-expression, number of shared interactions, shared GO-functions and conservation of the interaction at physical or genetic level in different species into a single likelihood score using a naive Bayesian approach (see Methods). The width of a line in Figure 5 is proportional to the likelihood score, were thicker lines represent more reliable predictions. From the 85 predicted interactions, 34 (40%) have more than 50% odds of being a true *in vivo* interaction in the face of this additional evidence. We can conclude that the predicted network is enriched for interactions strongly supported by experimental evidences. It is important to note that the quality of the predictions does not appear to be homogeneous, with some domains faring better than others. In particular we could not find supporting information for any of the predictions for the SH21A SH2 domain. This could be due the lack of information available for this protein and/or the poor performance of FoldX (Table 1) for its unconventional mode of binding [15].

We have investigated further some of the predicted novel interactions by compiling relevant information from the literature. For some of the interactions we could find either evidence for association (not currently annotated in HPRD) or we found supporting information from homologous proteins. For example, the Lck SH2 domain (LCK_HUMAN) has been shown to bind IRS1 (IRS1_HUMAN) peptides in vitro [52]. The expression of Lck was shown to be important for activation of Hematopoietic progenitor kinase (HPK1 or M4K1_HUMAN) and determinant for the efficient recruitment of HPK1 to the contact site of antigen-presenting T-cell conjugates [53]. It is possible that the predicted interaction between Lck and HPK1 might be important for this membrane recruitment. The predicted interaction between N-terminal SH2 domain of p85 regulatory subunit (P85A_HUMAN) and the fibroblast growth factor receptor 1 (FGFR1_HUMAN) has previously been shown by yeast-two-hybrid [54]. Also, the same interaction has been observed *in vivo* in *Xenopus blastulae* [55] and the injection of p85 alpha N-SH2 in *Xenopus laevis* oocytes was shown to impair FGFR1 signaling [55]. Our method also predicts that p85 interacts with FGFR2 and FGFR3 that by homology are also likely to be biologically relevant. These putative interactions emphasize the importance of the p85 N-SH2 for fibroblast receptor signaling.

Some of the putative interacting proteins form complexes with common targets that might hint at the biological roles of the predicted interactions. For example, both Wiskott-Aldrich syndrome like protein (WASL_HUMAN) and SAP (SH21A_HUMAN) have been shown to interact with an activated form of Cdc42 [56],[57]. WASL phosphorylation at tyrosine 253 can activate *in vitro* WASL-Arp2/3 actin polymerization in synergy with Cdc42-WASL interaction [57]. The predicted interaction between SAP-SH2 domain and phosphorylated Y253 of WASL may further enhance this synergistic effect *in vivo* by directing the activated form of Cdc42 to WASL.

This initial investigation of the possible biological functions of the predicted interactions further indicates that the predictions presented can be used to derive biologically relevant testable hypothesis.

## Discussion

We have implemented phosphorylation into FoldX in order to predict the binding specificity of SH2 domains. There are only nine available X-ray structures of human SH2 domains in complex with a target phosphopeptide (Table 1). We tried to overcome the modest size of this dataset by testing whether the upgraded version of FoldX can predict six different kinds of experimental results: the effect of dephosphorylation (Table S1) and of mutations in the environment of the peptide group on the stability of the complex (Figure 1), the consensus target sequence for the domain (Table 1), the *in vitro* binding specificity (Figure 2) and how it changes upon mutation of the SH2 domain (Table 2) and *in vivo* SH2-mediated protein-protein interactions (Figure 3). The performance of FoldX is comparable in all cases to consensus target sequences and substitution matrices derived from experiment. Based on this combination of results, we propose that the FoldX algorithm is a useful alternative to peptide library experiments for the prediction of SH2-mediated protein-protein interactions.

Several groups have developed other structure-based methods to predict the *in vitro* binding specificity of SH2 domains [20]–[22]. Henriques and coworkers tested several solvent-accessible surface area-based energy functions for binding of 6 phosphopeptides to the src SH2 domain, finding a poor correlation between their calculations and experiment [20]. Later algorithms using molecular dynamics [21] and comparative molecular field analysis [22] showed a good predictive power. Both studies focused on a small number of complexes (9 and 30, respectively) and on a single SH2 domain. The portability of these methods to other SH2 domains remains to be shown. Here, we have shown that FoldX can predict the in vitro binding thermodynamics of protein-phosphopeptide complexes including nine different SH2 domains, showing its applicability for genome-wide predictions.

The structure-based prediction of SH2-mediated *in vivo* protein-protein interactions has been addressed only once. McLaughlin and coworkers predicted the binding specificity of the Grb2 and SAP SH2 domains by combining information on known binding peptides plus structure-based predictions [23]. The resulting hidden Markov models gave predictions enriched in known in vivo interactions and binding sites [23]. The main advantage of our approach is that we derived the binding specificity using only structure-based calculations. Thus, our method is applicable to domains for which no binding experiments are available. We also benefit from the use of extra information on phosphorylation and secondary structure, which is available in databases or readily calculated from sequence.

Our approach is limited by the number of known structures of protein-phosphopeptide complexes. Nevertheless, given the structure of an SH2 domain in isolation, the location of the phosphopeptide binding site and the structure of a given SH2-phosphopeptide complex can be predicted computationally [58]-[60]. This, together with available methods for homology modelling of globular domains will widen the applicability of FoldX considerably in the near future.

FoldX can predict *in vivo* interactions as well as methods based on experimentally determined *in vitro* specificity. The fact that both methods based on binding specificity can make useful predictions confirms that specificity plays an important role in determining SH2-mediated protein interactions *in vivo*. On the other hand, the observed limitations of these methods strongly suggests that high affinity between an SH2 domain and its binding site is necessary but not sufficient to mediate binding *in vivo* due to other factors like co-expression, co-localization, phosphorylation and binding site availability requirements. FoldX may miss an SH2-mediated interaction in which specificity plays only a minor role, stressing the importance of integrating biological information into our method. We believe that future prediction methods should account for both the biophysics and the biology of SH2 domains.

We have used FoldX to predict *in vivo* protein-protein interactions for nine SH2 domain-containing proteins and annotated the predicted interactions with supporting information, providing a resource for further experimental testing. The predicted interactions are more informative than typical high-throughput or bioinformatics experiments in the sense that they provide binding site information and a structural template for the putative complex. Together with the prediction of binding specificity for other peptide binding domains and enzymes, we propose that FoldX can be used for the large-scale prediction and study of protein-protein interaction networks and signaling cascades and the impact of genetic variation in binding.

## Methods

### Parameterization of Phosphorylated Residues in FoldX

Proteins are represented in the algorithm as collections of residues and atoms with certain properties [24]. The main chain entropy and the properties of atoms not belonging to the phosphate moiety in pSer, pThr and pTyr were set to the corresponding values of serine, threonine and tyrosine. Similarly, the parameters of atoms belonging to the phosphate moiety were set to be the same for pSer, pThr and pTyr. Side chain entropy values were calculated by adding R·ln(6) to the values for the unphosphorylated residues, where six is the additional number of states for the phosphate group [24],[61]. Atom radii and volumes come from crystal structures of small compounds and the Voronoi analysis of structures of protein-nucleic acid complexes [62]. Van der Waals energies for the atoms in the phosphate groupwere calculated using the atomic volumes and a proportionality constant of −0.082 kcal/mol·Å^3^ [63]. The pK-values for the phosphate hydrogens in pSer, pThr and pTyr are at around 2 and 5.9 [64]. The pK-value for the second ionization can be significantly lower when interactions with other molecules are present [65]. Therefore, around neutral pH the charge of the phosphate group should be close to −2. We chose a charge of −0.60 for the oxygen atom in the phosphate group, which corresponds to a total charge for the phosphate moiety of −1.80. The average value of the solvation energy for charged atoms in FoldX is 3.33 kcal/mol per unit of charge [63]. We used this value to estimate a solvation energy of 2 kcal/mol per oxygen atom of the phosphate group.

### Calculation of Binding Energies

SH2 domains are globular and target phosphorylated motifs within disordered regions. Upon binding, these motifs adopt a single conformation, amenable to structure determination and FoldX calculations. We take into account folding-upon-binding by doing a stepwise calculation. First, we calculate the free energy for folding of the phosphopeptide into the conformation observed in the SH2-phosphopeptide complex (“folding energy”). Second, we calculate the free energy of interaction between the protein and the phosphopeptide in the complex (“interaction energy”). Last, we add the two numbers to calculate the free energy for formation of the protein-phosphopeptide complex (“binding energy”).

Substitution matrices are calculated as follows. First, the geometry of the wild-type complex was optimized. After this, we introduced all 20 residues at each phosphopeptide position. The “binding energy” for each residue at each position relative to the amino acid at the same position of the crystallized ligand was stored in a scoring matrix. The binding energy of a given sequence was calculated by summing over all positions of the matrix, which are taken to be independent.

Predictions using experimentally derived scoring matrices were obtained from the Scansite webserver (http://scansite.mit.edu/). It is not possible to obtain predicted binding scores covering the full dynamic range of the matrices from this web service so the lowest available threshold was selected. In the ROC curves calculated from Scansite predictions (Figures 2 and 3) the dotted line marks the threshold limit.

### Conservation and Phosphorylation Filters

We have previously shown that it is possible to improve the prediction of protein-interactions by combining the in-vitro binding specificity encoded in the form of linear motifs with additional information like conservation and secondary structure [51]. In this study, we compiled information on known phosphorylated tyrosines in the human proteome from the Human Protein Reference Database, Phosida and Phospho.ELM [48]–[50]. To these experimentally determined phosphorylation sites we added phospho-tyrosines predicted using the disPhos algorithm [47]. We also looked for the conservation of a putative binding site within a human protein in predicted orthologs in 9 other species (*Anopheles gambiae*, *Apis mellifera*, *Caenorhabditis elegans*, *Canis familiaris*, *Danio rerio*, *Fugu rubripes*, *Gallus gallus*, *Mus musculus*, *Pan troglodytes*). These genomes were selected on basis of their availability and to cover a broad evolutionary time scale of divergence from human. The ortholog assignments were taken from the Inparanoid database [66]. We considered that a putative binding site was conserved in another species when the orthologous protein also contained a predicted binding site.

### Naive Bayes Predictor

We have used a naive Bayes predictor [27] similar to the developed by Rhodes and colleagues [67] to integrate available information on conserved interactions, co-expression, shared interacting partners and shared GO function into a likelihood for the interactions of two proteins (Text S1). Briefly, we have considered a group of 8235 of *in vivo* protein interactions, found in the Human Protein Reference Database [49] (downloaded on 27 February 2006), as our positive standard. We considered that a protein defined in GO as belonging to the plasma membrane is less likely to interact with proteins in the nucleus and defined a negative set from pairs of such proteins (2,663,352 negative interactions). Using the positive and negative dataset we determined how each type of evidence impacts the odds that a pair of proteins interact. Assuming that the datasets are conditionally independent the likelihood ratio can be calculated as the product of individual likelihood ratios [27]. The tables of likelihood ratios calculated for each evidence type as well as a more detailed description of the different evidences used can be found in [27] and Text S1.

## Supporting Information

Figure S1 Conservation as a filter for FoldX predictions of SH2-mediated protein-protein interactions. (A) ROC curves for FoldX predictions (AROC 0.79±0.02), filtered for conservation in one (AROC 0.81±0.02), two (AROC 0.82±0.02), three (AROC 0.82±0.02) and four genomes (AROC 0.77±0.03). (B) ROC curves for FoldX predictions filtered for phosphorylation/secondary structure (AROC 0.92±0.02), filtered also for conservation in one (AROC 0.92±0.02), two (AROC 0.92±0.02), three (AROC 0.91±0.02) and four genomes (AROC 0.86±0.02).
(0.09 MB DOC)

Table S1 Experimental and calculated changes in free energy for protein-phosphopeptide complex formation upon dephosphorylation.
(0.12 MB DOC)

Table S2 Experimental and calculated changes in free energy for protein-phosphopeptide complex formation for mutations in the environment of the phosphate group in protein-phosphopeptide complexes.
(0.12 MB DOC)

Table S3 Binding and non-binding phosphopeptides. For all SH2 domains with available x-ray structure we compiled a list of binding and non-binding peptides from the literature. We could not find significant number of known binding and non-binding peptides for the C-terminal SH2 domain of Syk.
(0.50 MB DOC)

Table S4 Area under the ROC curve (AROC) statistics for prediction of peptide binding and full protein targets for human SH2 domains using FoldX and the Scansite server.
(0.08 MB DOC)

Table S5 Known SH2-mediated protein-protein interactions and binding sites in human.
(0.16 MB DOC)

Table S6 High-confidence predictions of SH2-mediated protein-protein interactions.
(0.27 MB DOC)

Text S1 Supplementary methods.
(0.18 MB DOC)
