# Supplementary material for: Genome-Wide Prediction of SH2 Domain Targets Using Structural Information and the FoldX Algorithm
Source: PLoS Comput Biol. 2008 Apr 4;4(4):e1000052. doi: 10.1371/journal.pcbi.1000052 (PMC2271153; doi:10.1371/journal.pcbi.1000052)

**Figure S1:** Conservation as a filter for FoldX predictions of SH2-mediated protein-protein interactions. (A) ROC curves for FoldX predictions (AROC 0.79±0.02), filtered for conservation in one (AROC 0.81±0.02), two (AROC 0.82±0.02), three (AROC 0.82±0.02) and four genomes (AROC 0.77±0.03). (B) ROC curves for FoldX predictions filtered for phosphorylation/secondary structure (AROC 0.92±0.02), filtered also for conservation in one (AROC 0.92±0.02), two (AROC 0.92±0.02), three (AROC 0.91±0.02) and four genomes (AROC 0.86±0.02).


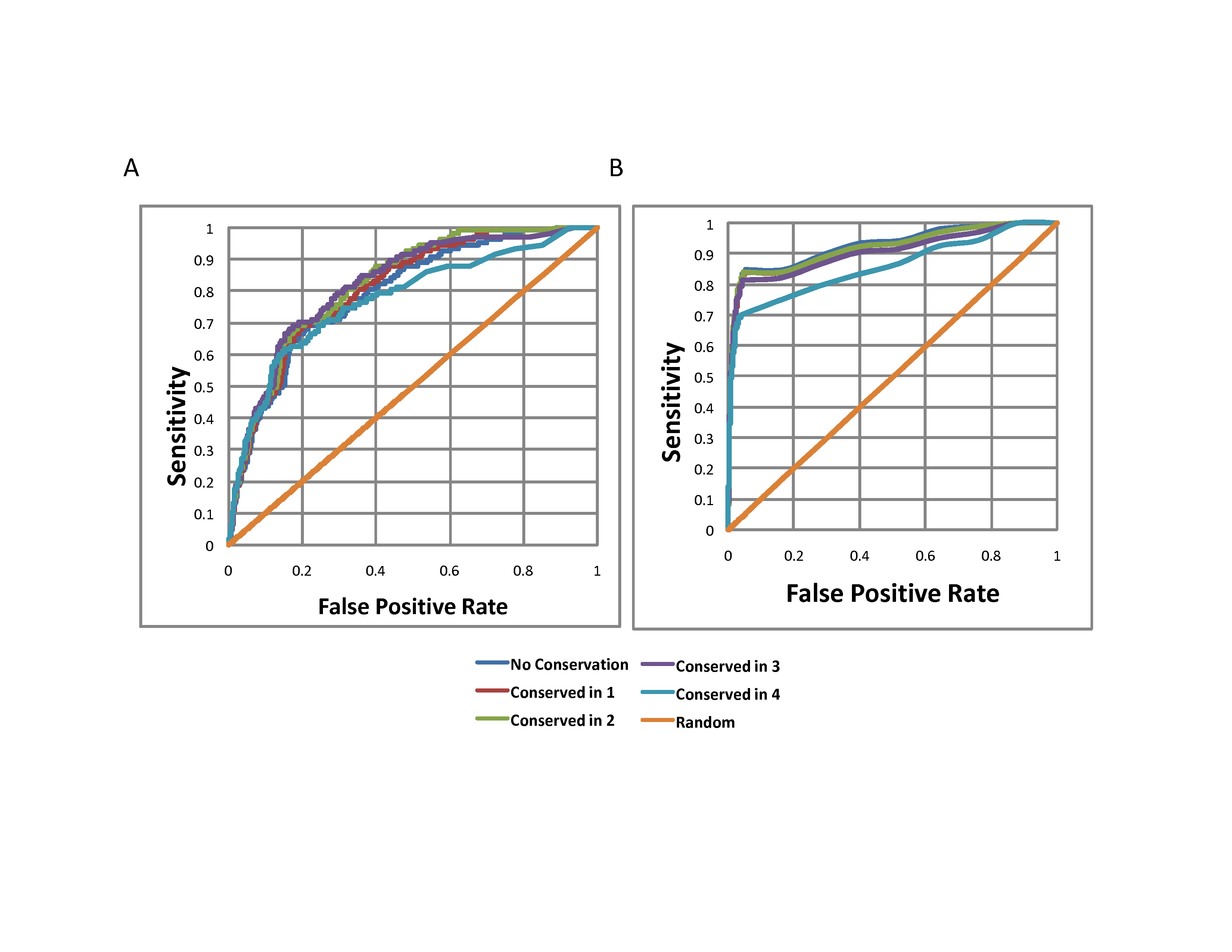

Supplement: Figure S1 — Conservation as a filter for FoldX predictions of SH2-mediated protein-protein interactions. (A) ROC curves for FoldX predictions (AROC 0.79±0.02), filtered for conservation in one (AROC 0.81±0.02), two (AROC 0.82±0.02), three (AROC 0.82±0.02) and four genomes (AROC 0.77±0.03). (B) ROC curves for FoldX predictions filtered for phosphorylation/secondary structure (AROC 0.92±0.02), filtered also for conservation in one (AROC 0.92±0.02), two (AROC 0.92±0.02), three (AROC 0.91±0.02) and four genomes (AROC 0.86±0.02). (0.09 MB DOC) [file pcbi.1000052.s001.doc]
