# Supplementary material for: Genome-Wide Prediction of SH2 Domain Targets Using Structural Information and the FoldX Algorithm
Source: PLoS Comput Biol. 2008 Apr 4;4(4):e1000052. doi: 10.1371/journal.pcbi.1000052 (PMC2271153; doi:10.1371/journal.pcbi.1000052)
Supplement: Table S1 — Experimental and calculated changes in free energy for protein-phosphopeptide complex formation upon dephosphorylation. (0.12 MB DOC) [file pcbi.1000052.s002.doc]

**Table S1:** Experimental and calculated changes in free energy for protein-phosphopeptide complex formation upon dephosphorylation.

| **Protein** | **Peptide** | **Structure** | **Resolution (Å)** | **Mutation** | **ΔΔG_dephosphorylation_ (kcal/mol)** | |
| --- | --- | --- | --- | --- | --- | --- |
|  |  |  |  |  | **Experimental** | **Calculated** |
| Pin1 full length | YpSPTpSPS | 1f8a | 1.84 | pS_1_ → S | 0.64 [1] | 1.63 |
| Pin1 full length | YpSPTpSPS | 1f8a | 1.84 | pS_2_ → S | 1.06 [1] | 3.86 |
| Pin1 WW domain | YpSPTpSPS | 1f8a | 1.84 | pS_1_ → S | 0.00 [1] | 1.66 |
| Pin1 WW domain | YpSPTpSPS | 1f8a | 1.84 | pS_2_ → S | 0.69 [1] | 3.92 |
| SCFCDC4 ubiquitin ligase | GLLpTPPQSG | 1nex | 2.70 | pT → pS | 1.06 [2] | 2.50 |
| GGA3 VHS domain | pSDEDLLHI | 1lf8 | 2.30 | pS → S | 0.69 [3] | 0.77 |
| GGA1 VHS domain | ADDIpSLLK | 1py1 | 2.60 | pS → S | 1.39 [4] | -0.10 |
| c-src SH2 domain | pYEEIE | 1shd | 2.00 | pY → Y | 5.60 [5] | 7.40 |
| Protein tyrosine phosphatase 1B | ETDpYpYR | 1g1f | 2.00 | pY_2_ → Y | 2.32 [6] | 3.05 |
| GCN5 | QTARKpSTGGKAPRKQLASK | 1pua | 2.30 | pS → S | 1.21 [7] | 0.28 |
| GGA2 VHS domain | DDIpSLLK | 1ujk | 1.90 | pS → S | 0.65 [4]h | -0.10 |
| Pin1 full length | YpSPTpSPS | 1f8a | 1.84 | pS_1_, pS_2_ → S | NB [1] | >3 |
| Pin1 WW domain | YpSPTpSPS | 1f8a | 1.84 | pS_1_, pS_2_ → S | NB [1] | >3 |
| SCFCDC4 ubiquitin ligase | GLLpTPPQSG | 1nex | 2.70 | pT → T | NB [2] | >3 |
| EAT-2 SH2 domain | SLTIpYAQVQK | 1i3z | 2.15 | pY → Y | NB [8] | >3 |
| DOK1 PTB domain | TWIENKLpYGM | 1uef | 2.50 | pY → Y | NB [9] | >3 |
| Protein tyrosine phosphatase 1B | DADEpYL | 1ptu | 2.60 | pY → Y | NB [10] | >3 |
| BRCA1 BRCT repeats | ISRSTpSPTFNKQ | 1t29 | 2.30 | pS → S | NB [11] | >3 |
| GADS SH2 domain | DDpYVNV | 1r1p | 1.80 | pY → Y | NB [12] | >3 |

**References**

1. Verdecia MA, Bowman ME, Lu KP, Hunter T, Noel JP (2000) Structural basis for phosphoserine-proline recognition by group IV WW domains. Nat Struct Biol 7: 639-643.

2. Nash P, Tang X, Orlicky S, Chen Q, Gertler FB, et al. (2001) Multisite phosphorylation of a CDK inhibitor sets a threshold for the onset of DNA replication. Nature 414: 514-521.

3. Kato Y, Misra S, Puertollano R, Hurley JH, Bonifacino JS (2002) Phosphoregulation of sorting signal-VHS domain interactions by a direct electrostatic mechanism. Nat Struct Biol 9: 532-536.

4. He X, Zhu G, Koelsch G, Rodgers KK, Zhang XC, et al. (2003) Biochemical and structural characterization of the interaction of memapsin 2 (beta-secretase) cytosolic domain with the VHS domain of GGA proteins. Biochemistry 42: 12174-12180.

5. Bradshaw JM, Mitaxov V, Waksman G (1999) Investigation of phosphotyrosine recognition by the SH2 domain of the Src kinase. J Mol Biol 293: 971-985.

6. Salmeen A, Andersen JN, Myers MP, Tonks NK, Barford D (2000) Molecular basis for the dephosphorylation of the activation segment of the insulin receptor by protein tyrosine phosphatase 1B. Mol Cell 6: 1401-1412.

7. Clements A, Poux AN, Lo WS, Pillus L, Berger SL, et al. (2003) Structural basis for histone and phosphohistone binding by the GCN5 histone acetyltransferase. Mol Cell 12: 461-473.

8. Morra M, Lu J, Poy F, Martin M, Sayos J, et al. (2001) Structural basis for the interaction of the free SH2 domain EAT-2 with SLAM receptors in hematopoietic cells. Embo J 20: 5840-5852.

9. Shi N, Ye S, Bartlam M, Yang M, Wu J, et al. (2004) Structural basis for the specific recognition of RET by the Dok1 phosphotyrosine binding domain. J Biol Chem 279: 4962-4969.

10. Milarski KL, Zhu G, Pearl CG, McNamara DJ, Dobrusin EM, et al. (1993) Sequence specificity in recognition of the epidermal growth factor receptor by protein tyrosine phosphatase 1B. J Biol Chem 268: 23634-23639.

11. Yu X, Chini CC, He M, Mer G, Chen J (2003) The BRCT domain is a phospho-protein binding domain. Science 302: 639-642.

12. Cho S, Velikovsky CA, Swaminathan CP, Houtman JC, Samelson LE, et al. (2004) Structural basis for differential recognition of tyrosine-phosphorylated sites in the linker for activation of T cells (LAT) by the adaptor Gads. Embo J 23: 1441-1451.
