# Supplementary material for: Genome-Wide Prediction of SH2 Domain Targets Using Structural Information and the FoldX Algorithm
Source: PLoS Comput Biol. 2008 Apr 4;4(4):e1000052. doi: 10.1371/journal.pcbi.1000052 (PMC2271153; doi:10.1371/journal.pcbi.1000052)
Supplement: Table S2 — Experimental and calculated changes in free energy for protein-phosphopeptide complex formation for mutations in the environment of the phosphate group in protein-phosphopeptide complexes. (0.12 MB DOC) [file pcbi.1000052.s003.doc]

**Table S2:** Experimental and calculated changes in free energy for protein-phosphopeptide complex formation for mutations in the environment of the phosphate group in protein-phosphopeptide complexes.

| **Protein** | **Peptide** | **Structure** | **Resolution (Å)** | **Mutation** | **Contacts with the phosphorylated residue** | **ΔΔG_dephosphorylation_ (kcal/mol)** | |
| --- | --- | --- | --- | --- | --- | --- | --- |
|  |  |  |  |  |  | **Experimental** | **Calculated** |
| 14-3-3 Z | RLYHpSLPA | 1qja | 2.00 | E180K | Ion pair | 0.92 [1] | 2.55 |
| P56-LCK SH2/SH3 domain | EGQpYQPQPA | 1lck | 2.50 | R134K | H-bond, cation-π interaction | 0.43 [2] | 0.25 |
| PLK-1 Polo-Box domain | PMQSpTPL | 1umw | 1.90 | H538A/K540M | H-bond/H-bond | 3.44 [3] | 2.45 |
| SAP SH2 domain | TIpYAQVQK | 1d4w | 1.80 | R32Q | Two H-bonds | 1.14 [4] | 2.23 |
| SAP SH2 domain | TIpYAQVQK | 1d4w | 1.80 | C42W | H-bond | 1.44 [4] | 0.17 |
| SAP SH2 domain | TIpYAQVQK | 1d4w | 1.80 | T53I | Hydrophobic contact | -0.02 [4] | 0.63 |
| Src SH2 domain | PQpYEpYIPA | 1nzl | 1.90 | R311A | H-bond | 0.53 [5] | 0.59 |
| Src SH2 domain | PQpYEpYIPA | 1nzl | 1.90 | R311F | H-bond | 0.00 [5] | 0.89 |
| Src SH2 domain | PQpYIpYVPA | 1nzv | 2.10 | R311A | H-bond | 0.90 [5] | 0.37 |
| Src SH2 domain | PQpYIpYVPA | 1nzv | 2.10 | R311F | H-bond | 0.00 [5] | 0.74 |
| Src SH2 domain | PQpYEEIP | 1sps | 2.70 | R12A | H-bond | 1.06 [6] | 1.69 |
| Src SH2 domain | PQpYEEIP | 1sps | 2.70 | R32A | Two H-bonds | 3.20 [6] | 2.87 |
| Src SH2 domain | PQpYEEIP | 1sps | 2.70 | S34A | H-bond | 0.89 [6] | 1.96 |
| Src SH2 domain | PQpYEEIP | 1sps | 2.70 | E35A | Ion pair | 0.43 [6] | -1.40 |
| Src SH2 domain | PQpYEEIP | 1sps | 2.70 | T36A | H-bond | 1.02 [6] | 0.68 |
| Src SH2 domain | PQpYEEIP | 1sps | 2.70 | T37A | H-bond network | -0.19 [6] | -0.06 |
| Src SH2 domain | PQpYEEIP | 1sps | 2.70 | C42A | Hydrophobic contact | -1.13 [6] | -0.04 |
| Src SH2 domain | PQpYEEIP | 1sps | 2.70 | C42S | Hydrophobic contact | -0.79 [6] | 0.22 |
| Src SH2 domain | PQpYEEIP | 1sps | 2.70 | S44A | H-bond network | 0.19 [6] | 0.09 |
| Src SH2 domain | PQpYEEIP | 1sps | 2.70 | H58A | H-bond network | 0.32 [6] | 1.08 |
| Src SH2 domain | PQpYEEIP | 1sps | 2.70 | K60A | H-bond, hydrophobic contact | 1.37 [6] | 1.11 |

**References**

1. Rittinger K, Budman J, Xu J, Volinia S, Cantley LC, et al. (1999) Structural analysis of 14-3-3 phosphopeptide complexes identifies a dual role for the nuclear export signal of 14-3-3 in ligand binding. Mol Cell 4: 153-166.

2. Lemmon MA, Ladbury JE (1994) Thermodynamic studies of tyrosyl-phosphopeptide binding to the SH2 domain of p56lck. Biochemistry 33: 5070-5076.

3. Elia AE, Rellos P, Haire LF, Chao JW, Ivins FJ, et al. (2003) The molecular basis for phosphodependent substrate targeting and regulation of Plks by the Polo-box domain. Cell 115: 83-95.

4. Hwang PM, Li C, Morra M, Lillywhite J, Muhandiram DR, et al. (2002) A "three-pronged" binding mechanism for the SAP/SH2D1A SH2 domain: structural basis and relevance to the XLP syndrome. Embo J 21: 314-323.

5. Lubman OY, Waksman G (2003) Structural and thermodynamic basis for the interaction of the Src SH2 domain with the activated form of the PDGF beta-receptor. J Mol Biol 328: 655-668.

6. Bradshaw JM, Mitaxov V, Waksman G (1999) Investigation of phosphotyrosine recognition by the SH2 domain of the Src kinase. J Mol Biol 293: 971-985.
