# Supplementary material for: Genome-Wide Prediction of SH2 Domain Targets Using Structural Information and the FoldX Algorithm
Source: PLoS Comput Biol. 2008 Apr 4;4(4):e1000052. doi: 10.1371/journal.pcbi.1000052 (PMC2271153; doi:10.1371/journal.pcbi.1000052)
Supplement: Table S3 — Binding and non-binding phosphopeptides. For all SH2 domains with available x-ray structure we compiled a list of binding and non-binding peptides from the literature. We could not find significant number of known binding and non-binding peptides for the C-terminal SH2 domain of Syk. (0.50 MB DOC) [file pcbi.1000052.s004.doc]

**Table S3: Binding and non-binding phosphopeptides. For all SH2 domains with available x-ray structure we compiled a list of binding and non-binding peptides from the literature. We could not find significant number of known binding and non-binding peptides for the C-terminal SH2 domain of Syk.**

| **SH2 domain** | **Binding information** | **Peptide tested experimentally** | **Peptide scored with FoldX** |
| --- | --- | --- | --- |
| Grb2 | Binding | ASyENE | SyENE |
| Grb2 | Binding | PCTGDyMNMSP | DyMNM |
| Grb2 | Binding | EPFyVNDPD | FyVND |
| Grb2 | Binding | AHPLNyVRRQFH | NyVRR |
| Grb2 | Binding | EPFyMNWPD | FyMNW |
| Grb2 | Binding | SDyINANI | DyINA |
| Grb2 | Binding | DDyVNV | DyVNV |
| Grb2 | Binding | REyVNV | EyVNV |
| Grb2 | Binding | SKAQEyFNIKSRSE | EyFNI |
| Grb2 | Binding | PVPyINQSVPKRK | PyINQ |
| Grb2 | Binding | TKASIyHRyHR | IyHRy |
| Grb2 | Binding | PLTCSPQPEyVNQPDVR | EyVNQ |
| Grb2 | Binding | DPSyVNVQ | SyVNV |
| Grb2 | Binding | WASYIyEQHARR | IyEQH |
| Grb2 | Binding | ADGMAyLNAKK | AyLNA |
| Grb2 | Binding | EPFyENVPD | FyENV |
| Grb2 | Binding | TGSEEyMNMDL | EyMNM |
| Grb2 | Binding | AYRNRyRRQYRY | RyRRQ |
| Grb2 | Binding | ATyVNVKS | TyVNV |
| Grb2 | Binding | VVHSGyRHQVPS | GyRHQ |
| Grb2 | Binding | SYMAPyDNYVP | PyDNY |
| Grb2 | Binding | PSAELySNALP | LySNA |
| Grb2 | Binding | DDyHNV | DyHNV |
| Grb2 | Binding | QRDVYYyERDMA | YyERD |
| Grb2 | Binding | EPFyVNVDD | FyVNV |
| Grb2 | Binding | KSPGEyVNIEF | EyVNI |
| Grb2 | Binding | EPFyENDPF | FyEND |
| Grb2 | Binding | GVDyKNI | DyKNI |
| Grb2 | Binding | EPQyENPPIYLK | QyENP |
| Grb2 | Binding | EPFyQNVPD | FyQNV |
| Grb2 | Binding | FSYKRyHPNRNH | RyHPN |
| Grb2 | Binding | DDyVNP | DyVNP |
| Grb2 | Binding | GHEyTNI | EyTNI |
| Grb2 | Binding | EPFyVNWPD | FyVNW |
| Grb2 | Binding | EPFyENWPD | FyENW |
| Grb2 | Binding | PQyQNyS | QyQNy |
| Grb2 | Binding | LFDDPSyVNYQN | SyVNY |
| Grb2 | Binding | PEKAKKAFDNPDyWNHSLPP | DyWNH |
| Grb2 | Binding | DHQyyND | QyyND |
| Grb2 | Binding | EPFyVtWPD | FyVtW |
| Grb2 | Binding | ARLEYyENEKK | YyENE |
| Grb2 | Binding | PDyENL | DyENL |
| Grb2 | Non-binding | TAGTTPDEDyEyMNRQRDG | DyEyM |
| Grb2 | Non-binding | TEEEDEDEEyEYMNRRR | EyEYM |
| Grb2 | Non-binding | FGGAVENPEyLTPQGGA | EyLTP |
| Grb2 | Non-binding | TDSAFDNPDyWHSRLFP | DyWHS |
| Grb2 | Non-binding | RPSSLEELGyEyMDVGSDL | GyEyM |
| Grb2 | Non-binding | AFSPAFDNLYyWDQDPPE | YyWDQ |
| Grb2 | Non-binding | LPSPTDSNFyRALMDEE | FyRAL |
| Grb2 | Non-binding | GACPASEQGyEEMRAFQ | GyEEM |
| Grb2 | Non-binding | MDDVVDADEyLIPQQGF | EyLIP |
| Grb2 | Non-binding | DDTFLPVPEyINQSVPK | EyINQ |
| Grb2 | Non-binding | PRSTLQHPDyLQEYSTK | DyLQE |
| Grb2 | Non-binding | GTPTAENPEyLGLDVPV | EyLGL |
| Grb2 | Non-binding | RPIVAENPEyLSEFSLK | EyLSE |
| Grb2 | Non-binding | ELMTFGSKPyDGIPASE | PyDGI |
| Grb2 | Non-binding | RPSSLEELGYEyMDVGSDL | EyMDV |
| Grb2 | Non-binding | MGDLVDAEEyLVPQQGF | EyLVP |
| Grb2 | Non-binding | DLQALDNPEyHNASNGP | EyHNA |
| Grb2 | Non-binding | AFSPAFDNLyyWDQDPPE | LyyWD |
| Grb2 | Non-binding | PMRDKPKQEyLNPVEEN | EyLNP |
| Grb2 | Non-binding | PICTIDVyMIMVK | VyMIM |
| Grb2 | Non-binding | KLLGAEEKEyHAEGGKV | EyHAE |
| Grb2 | Non-binding | TAGTTPDEDyEYMNRQR | DyEYM |
| Grb2 | Non-binding | RDGGGPGGDyAAMGACP | DyAAM |
| Grb2 | Non-binding | RPSSLEELGyEYMDVGS | GyEYM |
| Grb2 | Non-binding | AFSPAFDNLyYWDQDPPE | LyYWD |
| Grb2 | Non-binding | TEEEDEDEEyEyMNRRRRH | EyEyM |
| Grb2 | Non-binding | HSTAVGNPEyLNTVQPT | EyLNT |
| Grb2 | Non-binding | TAGTTPDEDYEyMNRQRDG | EyMNR |
| Grb2 | Non-binding | IKEDSFLQRySSDPTGA | RySSD |
| Grb2 | Non-binding | KGSTAENAEyLRVAPQS | EyLRV |
| Grb2 | Non-binding | NPAPSRDPHyQDPHSTA | HyQDP |
| Grb2 | Non-binding | HQISLDNPDyQQDFFPK | DyQQD |
| Grb2 | Non-binding | LPPDDKQLLySEAKTPI | LySEA |
| Grb2 | Non-binding | QSLLSPSSGyMPMNQGN | GyMPM |
| Grb2 | Non-binding | PAGSVQNPVyHNQPLNP | VyHNQ |
| LCK | Binding | TDSyAEIID | SyAEII |
| LCK | Binding | TATEGQyQPGP | QyQPGP |
| LCK | Binding | EPQyEELPIYL | QyEELP |
| LCK | Binding | EPQyEEEPIYL | QyEEEP |
| LCK | Binding | TATEGQyQPQP | QyQPQP |
| LCK | Binding | PEGDyEEVL | DyEEVL |
| LCK | Binding | VDGKEIyNTIRRK | IyNTIR |
| LCK | Binding | EPQyEEINIYL | QyEEIN |
| LCK | Binding | VSETDDyAEIIDE | DyAEII |
| LCK | Binding | IGSyIERDVTG | SyIERD |
| LCK | Binding | ESEyGNITYPPAMK | EyGNIT |
| LCK | Binding | EPQyEEQPIYL | QyEEQP |
| LCK | Binding | CVSyVVPTKAD | SyVVPT |
| LCK | Binding | GDGyMPMSPKS | GyMPMS |
| LCK | Binding | LGRREEyDVLDKR | EyDVLD |
| LCK | Binding | EPQyEEVPIYL | QyEEVP |
| LCK | Binding | EGQyEEGP | QyEEGP |
| LCK | Binding | EPQyEEIPIYL | QyEEIP |
| LCK | Binding | EPQyEIIPIYL | QyEIIP |
| LCK | Binding | EPQyQPGENL | QyQPGE |
| LCK | Binding | EPQyEAIPIYL | QyEAIP |
| LCK | Binding | EGQyEGIP | QyEGIP |
| LCK | Binding | ASQVyFTYDySE | VyFTYD |
| LCK | Binding | NHIySNLANSS | IySNLA |
| LCK | Binding | QGQNQLyNELNLG | LyNELN |
| LCK | Binding | EGPyEEIPIYL | PyEEIP |
| LCK | Binding | EPQyEEMPIYL | QyEEMP |
| LCK | Binding | EGQyGEIP | QyGEIP |
| LCK | Binding | GGVDyKNIHLE | DyKNIH |
| LCK | Non-binding | TAGTTPDEDYEyMNRQRDG | EyMNRQ |
| LCK | Non-binding | FGGAVENPEyLTPQGGA | EyLTPQ |
| LCK | Non-binding | MDDVVDADEyLIPQQGF | EyLIPQ |
| LCK | Non-binding | NPAPSRDPHyQDPHSTA | HyQDPH |
| LCK | Non-binding | LPPDDKQLLySEAKTPI | LySEAK |
| LCK | Non-binding | LPSPTDSNFyRALMDEE | FyRALM |
| LCK | Non-binding | IKEDSFLQRySSDPTGA | RySSDP |
| LCK | Non-binding | TAGTTPDEDyEYMNRQR | DyEYMN |
| LCK | Non-binding | DLQALDNPEyHNASNGP | EyHNAS |
| LCK | Non-binding | PAGSVQNPVyHNQPLNP | VyHNQP |
| LCK | Non-binding | DDTFLPVPEyINQSVPK | EyINQS |
| LCK | Non-binding | AFSPAFDNLYyWDQDPPE | YyWDQD |
| LCK | Non-binding | RPSSLEELGyEYMDVGS | GyEYMD |
| LCK | Non-binding | GACPASEQGyEEMRAFQ | GyEEMR |
| LCK | Non-binding | AFSPAFDNLyyWDQDPPE | LyyWDQ |
| LCK | Non-binding | HQISLDNPDyQQDFFPK | DyQQDF |
| LCK | Non-binding | QSLLSPSSGyMPMNQGN | GyMPMN |
| LCK | Non-binding | RPSSLEELGYEyMDVGSDL | EyMDVG |
| LCK | Non-binding | TEEEDEDEEyEYMNRRR | EyEYMN |
| LCK | Non-binding | TAGTTPDEDyEyMNRQRDG | DyEyMN |
| LCK | Non-binding | PLTCSPQPEyVNQPDVR | EyVNQP |
| LCK | Non-binding | KGSTAENAEyLRVAPQS | EyLRVA |
| LCK | Non-binding | TDSAFDNPDyWHSRLFP | DyWHSR |
| LCK | Non-binding | KLLGAEEKEyHAEGGKV | EyHAEG |
| LCK | Non-binding | AFSPAFDNLyYWDQDPPE | LyYWDQ |
| LCK | Non-binding | RPIVAENPEyLSEFSLK | EyLSEF |
| LCK | Non-binding | PMRDKPKQEyLNPVEEN | EyLNPV |
| LCK | Non-binding | NDSNyIVKGNA | NyIVKG |
| LCK | Non-binding | VDTyVEMRPVS | TyVEMR |
| LCK | Non-binding | GTPTAENPEyLGLDVPV | EyLGLD |
| LCK | Non-binding | LEKKyVRRDSG | KyVRRD |
| LCK | Non-binding | ELMTFGSKPyDGIPASE | PyDGIP |
| LCK | Non-binding | RDGGGPGGDyAAMGACP | DyAAMG |
| LCK | Non-binding | PRSTLQHPDyLQEYSTK | DyLQEY |
| LCK | Non-binding | HSTAVGNPEyLNTVQPT | EyLNTV |
| LCK | Non-binding | MGDLVDAEEyLVPQQGF | EyLVPQ |
| NCK1 | Binding | EEHIyDEVAADP | HIyDEVAAD |
| NCK1 | Binding | SSVLyTAVQPNE | VLyTAVQPN |
| NCK1 | Binding | FCPHyEKVSGDY | PHyEKVSGD |
| NCK1 | Binding | RDGGGPGGDyAAMGACP | GDyAAMGAC |
| NCK1 | Binding | MDDVVDADEyLIPQQGF | DEyLIPQQG |
| NCK1 | Binding | AFPGHLyDEVERT | HLyDEVERT |
| NCK1 | Binding | QDPRGIyDQVAGD | GIyDQVAGD |
| NCK1 | Binding | GAWGPLyDEVQMG | PLyDEVQMG |
| NCK1 | Binding | TEHIyDSVAGST | HIyDSVAGS |
| NCK1 | Non-binding | GTPTAENPEyLGLDVPV | PEyLGLDVP |
| NCK1 | Non-binding | KGSTAENAEyLRVAPQS | AEyLRVAPQ |
| NCK1 | Non-binding | DLQALDNPEyHNASNGP | PEyHNASNG |
| NCK1 | Non-binding | AFSPAFDNLyyWDQDPPE | NLyyWDQDP |
| NCK1 | Non-binding | DDTFLPVPEyINQSVPK | PEyINQSVP |
| NCK1 | Non-binding | TAGTTPDEDyEyMNRQRDG | EDyEyMNRQ |
| NCK1 | Non-binding | HQISLDNPDyQQDFFPK | PDyQQDFFP |
| NCK1 | Non-binding | MGDLVDAEEyLVPQQGF | EEyLVPQQG |
| NCK1 | Non-binding | PMRDKPKQEyLNPVEEN | QEyLNPVEE |
| NCK1 | Non-binding | AFSPAFDNLYyWDQDPPE | LYyWDQDPP |
| NCK1 | Non-binding | TAGTTPDEDYEyMNRQRDG | YEyMNRQRD |
| NCK1 | Non-binding | RPSSLEELGYEyMDVGSDL | YEyMDVGSD |
| NCK1 | Non-binding | QSLLSPSSGyMPMNQGN | SGyMPMNQG |
| NCK1 | Non-binding | RPSSLEELGyEYMDVGS | LGyEYMDVG |
| NCK1 | Non-binding | GACPASEQGyEEMRAFQ | QGyEEMRAF |
| NCK1 | Non-binding | PAGSVQNPVyHNQPLNP | PVyHNQPLN |
| NCK1 | Non-binding | AFSPAFDNLyYWDQDPPE | NLyYWDQDP |
| NCK1 | Non-binding | KLLGAEEKEyHAEGGKV | KEyHAEGGK |
| NCK1 | Non-binding | TAGTTPDEDyEYMNRQR | EDyEYMNRQ |
| NCK1 | Non-binding | NPAPSRDPHyQDPHSTA | PHyQDPHST |
| NCK1 | Non-binding | PLTCSPQPEyVNQPDVR | PEyVNQPDV |
| NCK1 | Non-binding | LPSPTDSNFyRALMDEE | NFyRALMDE |
| NCK1 | Non-binding | IKEDSFLQRySSDPTGA | QRySSDPTG |
| NCK1 | Non-binding | TDSAFDNPDyWHSRLFP | PDyWHSRLF |
| NCK1 | Non-binding | FGGAVENPEyLTPQGGA | PEyLTPQGG |
| NCK1 | Non-binding | LPPDDKQLLySEAKTPI | LLySEAKTP |
| NCK1 | Non-binding | RPIVAENPEyLSEFSLK | PEyLSEFSL |
| NCK1 | Non-binding | PRSTLQHPDyLQEYSTK | PDyLQEYST |
| NCK1 | Non-binding | TEEEDEDEEyEYMNRRR | EEyEYMNRR |
| NCK1 | Non-binding | HSTAVGNPEyLNTVQPT | PEyLNTVQP |
| NCK1 | Non-binding | ELMTFGSKPyDGIPASE | KPyDGIPAS |
| p85 | Binding | GNGDyMPMSPKS | NGDyMPMS |
| p85 | Binding | DGGyMDMSKDE | DGGyMDMS |
| p85 | Binding | SNQEyLDLSMPLDQ | NQEyLDLS |
| p85 | Binding | EEEyMPMEDLYL | EEEyMPME |
| p85 | Binding | ISLDAPDyQQDF | APDyQQDF |
| p85 | Binding | PNGyMMMSPSG | PNGyMMMS |
| p85 | Binding | GACPASEQGyEEMRAFQ | EQGyEEMR |
| p85 | Binding | SEEyMNMDLGP | SEEyMNMD |
| p85 | Binding | DNDyIIPLPDPK | DNDyIIPL |
| p85 | Binding | TGDyMNMSPVG | TGDyMNMS |
| p85 | Binding | RENEyMPMAPQIH | ENEyMPMA |
| p85 | Binding | GNGDSyMPMPKS | GDSyMPMP |
| p85 | Binding | SVDyVPMLDMK | SVDyVPML |
| p85 | Binding | VDTyVEMRPVS | VDTyVEMR |
| p85 | Binding | TNEyMDMKPGV | TNEyMDMK |
| p85 | Binding | DDGyMPMSPGV | DDGyMPMS |
| p85 | Binding | SVDyVDMSK | SVDyVDMS |
| p85 | Binding | LQGHIIENPQyFSDACVH | NPQyFSDA |
| p85 | Binding | QSLLSPSSGyMPMNQGN | SSGyMPMN |
| p85 | Binding | SSNyMAyDNY | SSNyMAyD |
| p85 | Binding | ISLDNADyQQDF | NADyQQDF |
| p85 | Non-binding | AFSPAFDNLyyWDQDPPE | DNLyyWDQ |
| p85 | Non-binding | TEEEDEDEEyEYMNRRR | DEEyEYMN |
| p85 | Non-binding | RPIVAENPEyLSEFSLK | NPEyLSEF |
| p85 | Non-binding | RDGGGPGGDyAAMGACP | GGDyAAMG |
| p85 | Non-binding | TAGTTPDEDyEYMNRQR | DEDyEYMN |
| p85 | Non-binding | GTPTAENPEyLGLDVPV | NPEyLGLD |
| p85 | Non-binding | TAGTTPDEDyEyMNRQRDG | DEDyEyMN |
| p85 | Non-binding | RPSSLEELGyEYMDVGS | ELGyEYMD |
| p85 | Non-binding | HQISLDNPDyQQDFFPK | NPDyQQDF |
| p85 | Non-binding | IKEDSFLQRySSDPTGA | LQRySSDP |
| p85 | Non-binding | LPSPTDSNFyRALMDEE | SNFyRALM |
| p85 | Non-binding | TDSAFDNPDyWHSRLFP | NPDyWHSR |
| p85 | Non-binding | AFSPAFDNLYyWDQDPPE | NLYyWDQD |
| p85 | Non-binding | DDTFLPVPEyINQSVPK | VPEyINQS |
| p85 | Non-binding | KGSTAENAEyLRVAPQS | NAEyLRVA |
| p85 | Non-binding | AFSPAFDNLyYWDQDPPE | DNLyYWDQ |
| p85 | Non-binding | PLTCSPQPEyVNQPDVR | QPEyVNQP |
| p85 | Non-binding | DADEyLIPQQGFFSK | ADEyLIPQ |
| p85 | Non-binding | NPAPSRDPHyQDPHSTA | DPHyQDPH |
| p85 | Non-binding | HSTAVGNPEyLNTVQPT | NPEyLNTV |
| p85 | Non-binding | EPQyQPGENL | EPQyQPGE |
| p85 | Non-binding | PRSTLQHPDyLQEYSTK | HPDyLQEY |
| p85 | Non-binding | DLQALDNPEyHNASNGP | NPEyHNAS |
| p85 | Non-binding | FGGAVENPEyLTPQGGA | NPEyLTPQ |
| p85 | Non-binding | LPPDDKQLLySEAKTPI | QLLySEAK |
| p85 | Non-binding | TAGTTPDEDYEyMNRQRDG | DYEyMNRQ |
| p85 | Non-binding | PMRDKPKQEyLNPVEEN | KQEyLNPV |
| p85 | Non-binding | RPSSLEELGYEyMDVGSDL | GYEyMDVG |
| p85 | Non-binding | PAGSVQNPVyHNQPLNP | NPVyHNQP |
| p85 | Non-binding | MGDLVDAEEyLVPQQGF | AEEyLVPQ |
| p85 | Non-binding | ELMTFGSKPyDGIPASE | SKPyDGIP |
| p85 | Non-binding | KLLGAEEKEyHAEGGKV | EKEyHAEG |
| SAP | Binding | GGSTIySMIQS | GSTIySMIQS |
| SAP | Binding | KSLTIyAQVQK | SLTIyAQVQK |
| SAP | Binding | PAYTLySLIQP | AYTLySLIQP |
| SAP | Binding | PVNTVySEVQF | VNTVySEVQF |
| SAP | Binding | DPVTPyVTEVE | PVTPyVTEVE |
| SAP | Binding | EFLTIyEDVKD | FLTIyEDVKD |
| SAP | Binding | SKKTIyTYIMA | KKTIyTYIMA |
| SAP | Binding | GSITVyASVTL | SITVyASVTL |
| SAP | Binding | GENTVyAQVFN | ENTVyAQVFN |
| SAP | Binding | PCTTIyVAATE | CTTIyVAATE |
| SAP | Binding | FNSTIyEVIGH | NSTIyEVIGH |
| SAP | Non-binding | MGDLVDAEEyLVPQQGF | DAEEyLVPQQ |
| SAP | Non-binding | TAGTTPDEDyEYMNRQR | PDEDyEYMNR |
| SAP | Non-binding | TEEEDEDEEyEYMNRRR | EDEEyEYMNR |
| SAP | Non-binding | RDGGGPGGDyAAMGACP | PGGDyAAMGA |
| SAP | Non-binding | KLLGAEEKEyHAEGGKV | EEKEyHAEGG |
| SAP | Non-binding | TDSAFDNPDyWHSRLFP | DNPDyWHSRL |
| SAP | Non-binding | AFSPAFDNLyyWDQDPPE | FDNLyyWDQD |
| SAP | Non-binding | PMRDKPKQEyLNPVEEN | PKQEyLNPVE |
| SAP | Non-binding | KGSTAENAEyLRVAPQS | ENAEyLRVAP |
| SAP | Non-binding | ELMTFGSKPyDGIPASE | GSKPyDGIPA |
| SAP | Non-binding | GACPASEQGyEEMRAFQ | SEQGyEEMRA |
| SAP | Non-binding | AFSPAFDNLyYWDQDPPE | FDNLyYWDQD |
| SAP | Non-binding | LPSPTDSNFyRALMDEE | DSNFyRALMD |
| SAP | Non-binding | QSLLSPSSGyMPMNQGN | PSSGyMPMNQ |
| SAP | Non-binding | RPSSLEELGYEyMDVGSDL | LGYEyMDVGS |
| SAP | Non-binding | PAGSVQNPVyHNQPLNP | QNPVyHNQPL |
| SAP | Non-binding | FGGAVENPEyLTPQGGA | ENPEyLTPQG |
| SAP | Non-binding | GTPTAENPEyLGLDVPV | ENPEyLGLDV |
| SAP | Non-binding | DDTFLPVPEyINQSVPK | PVPEyINQSV |
| SAP | Non-binding | PRSTLQHPDyLQEYSTK | QHPDyLQEYS |
| SAP | Non-binding | RPIVAENPEyLSEFSLK | ENPEyLSEFS |
| SAP | Non-binding | NPAPSRDPHyQDPHSTA | RDPHyQDPHS |
| SAP | Non-binding | PLTCSPQPEyVNQPDVR | PQPEyVNQPD |
| SAP | Non-binding | AFSPAFDNLYyWDQDPPE | DNLYyWDQDP |
| SAP | Non-binding | DLQALDNPEyHNASNGP | DNPEyHNASN |
| SAP | Non-binding | LPPDDKQLLySEAKTPI | KQLLySEAKT |
| SAP | Non-binding | TAGTTPDEDYEyMNRQRDG | EDYEyMNRQR |
| SAP | Non-binding | IKEDSFLQRySSDPTGA | FLQRySSDPT |
| SAP | Non-binding | HQISLDNPDyQQDFFPK | DNPDyQQDFF |
| SAP | Non-binding | MDDVVDADEyLIPQQGF | DADEyLIPQQ |
| SAP | Non-binding | RPSSLEELGyEYMDVGS | EELGyEYMDV |
| SAP | Non-binding | HSTAVGNPEyLNTVQPT | GNPEyLNTVQ |
| SAP | Non-binding | TAGTTPDEDyEyMNRQRDG | PDEDyEyMNR |
| SRC | Binding | EPQyEEEPIYL | PQyEEEP |
| SRC | Binding | PQyAEIPI | PQyAEIP |
| SRC | Binding | EPQyEEDPIYL | PQyEEDP |
| SRC | Binding | GDGyMPMSPKS | DGyMPMS |
| SRC | Binding | PQyRAIPI | PQyRAIP |
| SRC | Binding | EPQyQPGENL | PQyQPGE |
| SRC | Binding | ESEyGNITYPPAMK | SEyGNIT |
| SRC | Binding | PQyEAIPI | PQyEAIP |
| SRC | Binding | TEGQyQPQPA | GQyQPQP |
| SRC | Binding | EPQyEEIyL | PQyEEIy |
| SRC | Binding | EPQyEEIPIYL | PQyEEIP |
| SRC | Binding | EPQyENPPIYLK | PQyENPP |
| SRC | Binding | RDGGGPGGDyAAMGACP | GDyAAMG |
| SRC | Binding | PQyEEAPI | PQyEEAP |
| SRC | Binding | PQyREIPI | PQyREIP |
| SRC | Binding | GACPASEQGyEEMRAFQ | QGyEEMR |
| SRC | Binding | TSTEPQyEEIENL | PQyEEIE |
| SRC | Binding | PQyEEVPI | PQyEEVP |
| SRC | Binding | DPSyVNVQNLDK | PSyVNVQ |
| SRC | Binding | KGGQyEEIPIP | GQyEEIP |
| SRC | Binding | PQyKAIPI | PQyKAIP |
| SRC | Binding | GLSRyMEDST | SRyMEDS |
| SRC | Binding | VSETDDyAEIIDE | DDyAEII |
| SRC | Binding | PQyAAIPI | PQyAAIP |
| SRC | Binding | PQyEELPI | PQyEELP |
| SRC | Binding | PEGDyEEVL | GDyEEVL |
| SRC | Binding | TQyVPMLE | TQyVPML |
| SRC | Binding | DNDyEEFLPDPK | NDyEEFL |
| SRC | Binding | EDSTyyKASKG | STyyKAS |
| SRC | Binding | DGKEIyNTIRRK | EIyNTIR |
| SRC | Non-binding | PICTIDVyMIMVK | DVyMIMV |
| SRC | Non-binding | ELMTFGSKPyDGIPASE | KPyDGIP |
| SRC | Non-binding | KLLGAEEKEyHAEGGKV | KEyHAEG |
| SRC | Non-binding | PAGSVQNPVyHNQPLNP | PVyHNQP |
| SRC | Non-binding | TAGTTPDEDYEyMNRQRDG | YEyMNRQ |
| SRC | Non-binding | KGSTAENAEyLRVAPQS | AEyLRVA |
| SRC | Non-binding | AFSPAFDNLyYWDQDPPE | NLyYWDQ |
| SRC | Non-binding | LPSPTDSNFyRALMDEE | NFyRALM |
| SRC | Non-binding | AFSPAFDNLYyWDQDPPE | LYyWDQD |
| SRC | Non-binding | RPSSLEELGyEYMDVGS | LGyEYMD |
| SRC | Non-binding | FGGAVENPEyLTPQGGA | PEyLTPQ |
| SRC | Non-binding | PLTCSPQPEyVNQPDVR | PEyVNQP |
| SRC | Non-binding | DDTFLPVPEyINQSVPK | PEyINQS |
| SRC | Non-binding | RPSSLEELGyEyMDVGSDL | LGyEyMD |
| SRC | Non-binding | QSLLSPSSGyMPMNQGN | SGyMPMN |
| SRC | Non-binding | MDDVVDADEyLIPQQGF | DEyLIPQ |
| SRC | Non-binding | GTPTAENPEyLGLDVPV | PEyLGLD |
| SRC | Non-binding | NPAPSRDPHyQDPHSTA | PHyQDPH |
| SRC | Non-binding | RPSSLEELGYEyMDVGSDL | YEyMDVG |
| SRC | Non-binding | LPPDDKQLLySEAKTPI | LLySEAK |
| SRC | Non-binding | TAGTTPDEDyEyMNRQRDG | EDyEyMN |
| SRC | Non-binding | TEEEDEDEEyEYMNRRR | EEyEYMN |
| SRC | Non-binding | IKEDSFLQRySSDPTGA | QRySSDP |
| SRC | Non-binding | TEEEDEDEEYEyMNRRRRH | YEyMNRR |
| SRC | Non-binding | TEEEDEDEEyEyMNRRRRH | EEyEyMN |
| SRC | Non-binding | HSTAVGNPEyLNTVQPT | PEyLNTV |
| SRC | Non-binding | HQISLDNPDyQQDFFPK | PDyQQDF |
| SRC | Non-binding | TAGTTPDEDyEYMNRQR | EDyEYMN |
| SRC | Non-binding | MGDLVDAEEyLVPQQGF | EEyLVPQ |
| SRC | Non-binding | AFSPAFDNLyyWDQDPPE | NLyyWDQ |
| STAT1 | Binding | DPSyVNVQ | yVNVQ |
| STAT1 | Binding | TKASIyHRyHR | yHRyH |
| STAT1 | Binding | FSYKRyHPNRNH | yHPNR |
| STAT1 | Binding | PTSFGyDKPHVL | yDKPH |
| STAT1 | Binding | QERRKyLKHRLI | yLKHR |
| STAT1 | Binding | WASYIyEQHARR | yEQHA |
| STAT1 | Binding | RHVRTySPRHQY | ySPRH |
| STAT1 | Binding | VKYKDyKPQYAY | yKPQY |
| STAT1 | Binding | SNLNYyKPRQFT | yKPRQ |
| STAT1 | Binding | VVHSGyRHQVPS | yRHQV |
| STAT1 | Binding | VDYEYyERQHDY | yERQH |
| STAT1 | Binding | VSFNRyFHQNYP | yFHQN |
| STAT1 | Binding | KRYHSyRSRPRA | yRSRP |
| STAT1 | Binding | GKYQMyHRQPRA | yHRQP |
| STAT1 | Binding | LTSFGyRPRDRQ | yRPRD |
| STAT1 | Binding | QRDVYYyERDMA | yERDM |
| STAT1 | Binding | PKGTGyIKTELI | yIKTE |
| STAT1 | Binding | LGNIAyLPQHVN | yLPQH |
| STAT1 | Binding | AFSPAFDNLyYWDQDPPE | yYWDQ |
| STAT1 | Binding | GMPKSyLPQTVR | yLPQT |
| STAT1 | Binding | AFSPAFDNLYyWDQDPPE | yWDQD |
| STAT1 | Binding | HLEKKyVRRDSG | yVRRD |
| STAT1 | Binding | AYRNRyRRQYRY | yRRQY |
| STAT1 | Binding | AHPLNyVRRQFH | yVRRQ |
| STAT1 | Binding | VDYKYyDPRHDL | yDPRH |
| STAT1 | Binding | TKASIyHRPYHR | yHRPY |
| STAT1 | Binding | KARNPyISYKRF | yISYK |
| STAT1 | Non-binding | NPAPSRDPHyQDPHSTA | yQDPH |
| STAT1 | Non-binding | FGGAVENPEyLTPQGGA | yLTPQ |
| STAT1 | Non-binding | RPIVAENPEyLSEFSLK | yLSEF |
| STAT1 | Non-binding | RDGGGPGGDyAAMGACP | yAAMG |
| STAT1 | Non-binding | RPSSLEELGYEyMDVGSDL | yMDVG |
| STAT1 | Non-binding | QSLLSPSSGyMPMNQGN | yMPMN |
| STAT1 | Non-binding | AFSPAFDNLyyWDQDPPE | yyWDQ |
| STAT1 | Non-binding | DLQALDNPEyHNASNGP | yHNAS |
| STAT1 | Non-binding | GACPASEQGyEEMRAFQ | yEEMR |
| STAT1 | Non-binding | KLLGAEEKEyHAEGGKV | yHAEG |
| STAT1 | Non-binding | LPPDDKQLLySEAKTPI | ySEAK |
| STAT1 | Non-binding | ELMTFGSKPyDGIPASE | yDGIP |
| STAT1 | Non-binding | TAGTTPDEDyEYMNRQR | yEYMN |
| STAT1 | Non-binding | PAGSVQNPVyHNQPLNP | yHNQP |
| STAT1 | Non-binding | LPSPTDSNFyRALMDEE | yRALM |
| STAT1 | Non-binding | HSTAVGNPEyLNTVQPT | yLNTV |
| STAT1 | Non-binding | HQISLDNPDyQQDFFPK | yQQDF |
| STAT1 | Non-binding | MGDLVDAEEyLVPQQGF | yLVPQ |
| STAT1 | Non-binding | RPSSLEELGyEYMDVGS | yEYMD |
| STAT1 | Non-binding | PRSTLQHPDyLQEYSTK | yLQEY |
| STAT1 | Non-binding | TAGTTPDEDYEyMNRQRDG | yMNRQ |
| STAT1 | Non-binding | PLTCSPQPEyVNQPDVR | yVNQP |
| STAT1 | Non-binding | TAGTTPDEDyEyMNRQRDG | yEyMN |
| STAT1 | Non-binding | MDDVVDADEyLIPQQGF | yLIPQ |
| STAT1 | Non-binding | PMRDKPKQEyLNPVEEN | yLNPV |
| STAT1 | Non-binding | TDSAFDNPDyWHSRLFP | yWHSR |
| STAT1 | Non-binding | GTPTAENPEyLGLDVPV | yLGLD |
| STAT1 | Non-binding | DDTFLPVPEyINQSVPK | yINQS |
| STAT1 | Non-binding | KGSTAENAEyLRVAPQS | yLRVA |
| STAT1 | Non-binding | IKEDSFLQRySSDPTGA | ySSDP |
| SYK (N-term) | Binding | FGGAVENPEyLTPQGGA | ENPEyLTPQ |
| SYK (N-term) | Binding | LPPDDKQLLySEAKTPI | KQLLySEAK |
| SYK (N-term) | Binding | LPSPTDSNFyRALMDEE | DSNFyRALM |
| SYK (N-term) | Binding | MGDLVDAEEyLVPQQGF | DAEEyLVPQ |
| SYK (N-term) | Binding | RPIVAENPEyLSEFSLK | ENPEyLSEF |
| SYK (N-term) | Binding | AFSPAFDNLyYWDQDPPE | FDNLyYWDQ |
| SYK (N-term) | Binding | ELMTFGSKPyDGIPASE | GSKPyDGIP |
| SYK (N-term) | Binding | TEEEDEDEEyEYMNRRR | EDEEyEYMN |
| SYK (N-term) | Binding | NPAPSRDPHyQDPHSTA | RDPHyQDPH |
| SYK (N-term) | Binding | MDDVVDADEyLIPQQGF | DADEyLIPQ |
| SYK (N-term) | Binding | TAGTTPDEDyEYMNRQR | PDEDyEYMN |
| SYK (N-term) | Binding | PLTCSPQPEyVNQPDVR | PQPEyVNQP |
| SYK (N-term) | Binding | HQISLDNPDyQQDFFPK | DNPDyQQDF |
| SYK (N-term) | Binding | RPSSLEELGyEYMDVGS | EELGyEYMD |
| SYK (N-term) | Binding | AFSPAFDNLyyWDQDPPE | FDNLyyWDQ |
| SYK (N-term) | Binding | RDGGGPGGDyAAMGACP | PGGDyAAMG |
| SYK (N-term) | Binding | GACPASEQGyEEMRAFQ | SEQGyEEMR |
| SYK (N-term) | Binding | AFSPAFDNLYyWDQDPPE | DNLYyWDQD |
| SYK (N-term) | Non-binding | PMRDKPKQEyLNPVEEN | PKQEyLNPV |
| SYK (N-term) | Non-binding | PRSTLQHPDyLQEYSTK | QHPDyLQEY |
| SYK (N-term) | Non-binding | KLLGAEEKEyHAEGGKV | EEKEyHAEG |
| SYK (N-term) | Non-binding | KGSTAENAEyLRVAPQS | ENAEyLRVA |
| SYK (N-term) | Non-binding | TAGTTPDEDYEyMNRQRDG | EDYEyMNRQ |
| SYK (N-term) | Non-binding | TAGTTPDEDyEyMNRQRDG | PDEDyEyMN |
| SYK (N-term) | Non-binding | HSTAVGNPEyLNTVQPT | GNPEyLNTV |
| SYK (N-term) | Non-binding | PAGSVQNPVyHNQPLNP | QNPVyHNQP |
| SYK (N-term) | Non-binding | GTPTAENPEyLGLDVPV | ENPEyLGLD |
| SYK (N-term) | Non-binding | DDTFLPVPEyINQSVPK | PVPEyINQS |
| SYK (N-term) | Non-binding | IKEDSFLQRySSDPTGA | FLQRySSDP |
| SYK (N-term) | Non-binding | DLQALDNPEyHNASNGP | DNPEyHNAS |
| SYK (N-term) | Non-binding | RPSSLEELGYEyMDVGSDL | LGYEyMDVG |
| SYK (N-term) | Non-binding | TDSAFDNPDyWHSRLFP | DNPDyWHSR |
| SYK (N-term) | Non-binding | QSLLSPSSGyMPMNQGN | PSSGyMPMN |
