# Supplementary material for: Genome-Wide Prediction of SH2 Domain Targets Using Structural Information and the FoldX Algorithm
Source: PLoS Comput Biol. 2008 Apr 4;4(4):e1000052. doi: 10.1371/journal.pcbi.1000052 (PMC2271153; doi:10.1371/journal.pcbi.1000052)
Supplement: Table S4 — Area under the ROC curve (AROC) statistics for prediction of peptide binding and full protein targets for human SH2 domains using FoldX and the Scansite server. (0.08 MB DOC) [file pcbi.1000052.s005.doc]

**Table S4. Area under the ROC curve (AROC) statistics for prediction of peptide binding and full protein targets for human SH2 domains using FoldX and the Scansite server.**

N.T.: Testing was not possible for this domain for lack of sufficient target peptides or target proteins. N.S.: No predictor was available for this domain on the Scansite server. The AROC statistics were obtained using the SPSS statistics package under the nonparametric assumption and a confidence level of 95%. The asymptotic significance (Asymp. Sig.) refers to the null hypothesis of the true area being equal to 0.5.

|  | **FoldX peptide predictions** | | **Scansite peptide predictions** | | **FoldX protein predictions** | | **Scansite protein predictions** | |
| --- | --- | --- | --- | --- | --- | --- | --- | --- |
| **SH2 domain** | **AROC** | **Asymp. Sig.** | **AROC** | **Asymp. Sig.** | **AROC** | **Asymp. Sig.** | **AROC** | **Asymp. Sig.** |
| **Grb2** | 0.82±0.05 | 1.2⋅10^-6^ | 0.82±0.05 | 1.1⋅10^-6^ | 0.79±0.03 | 3.6⋅10^-9^ | 0.77±0.05 | 5.8⋅10^-8^ |
| **Sap** | 0.88±0.07 | 1.6⋅10^-4^ | N.S. | - | N.T. | - | N.S. | - |
| **p85** | 0.77±0.07 | 8.8⋅10^-4^ | 0.78±0.07 | 7.7⋅10^-4^ | 0.78±0.05 | 2.7⋅10^-4^ | 0.83±0.07 | 2.2⋅10^-5^ |
| **Src** | 0.64±0.07 | 0.06 | 0.63±0.07 | 0.09 | 0.84±0.05 | 5.6⋅10^-5^ | 0.66±0.10 | 0.06 |
| **Lck** | 0.63±0.07 | 0.07 | 0.62±0.07 | 0.10 | 0.76±0.05 | 1.5⋅10^-4^ | 0.76±0.07 | 1.7⋅10^-4^ |
| **Nck1** | 0.65±0.11 | 0.17 | 0.72±0.11 | 0.05 | 0.69±0.08 | 0.04 | 0.72±0.10 | 0.02 |
| **Syk (N-term)** | 0.60±0.10 | 0.31 | N.S. | - | 0.70±0.06 | 0.05 | N.S. | - |
| **Stat1** | 0.44±0.08 | 0.42 | N.S. | - | 0.69±0.13 | 0.25 | N.S | - |
| **Syk (C-term)** | N.T. | - | N.S./N.T. | - | 0.77±0.07 | 0.01 | N.S. | - |
