# Supplementary material for: Genome-Wide Prediction of SH2 Domain Targets Using Structural Information and the FoldX Algorithm
Source: PLoS Comput Biol. 2008 Apr 4;4(4):e1000052. doi: 10.1371/journal.pcbi.1000052 (PMC2271153; doi:10.1371/journal.pcbi.1000052)
Supplement: Table S5 — Known SH2-mediated protein-protein interactions and binding sites in human. (0.16 MB DOC) [file pcbi.1000052.s006.doc]

**Table S5:** **Known SH2-mediated protein-protein interactions and binding sites in human**. Experimentally determined interactions for p85 (ENSP00000274335), SYK (ENSP00000326032), STAT1 (ENSP00000354394), LCK (ENSP00000328213), SRC (ENSP00000353950) ,GRB (ENSP00000339007) and NCK1 (ENSP00000288986) were taken from the Human Protein Reference Database (16/06/2006) and were manually curated in order to identify interactions mediated by an SH2 domain and, whenever possible the exact binding site within the target protein. We kept only binding site information that we could confidently match to the protein sequences used. The 95 interactions found are listed below, annotated with their Ensembl protein IDs, experimental evidence type and pubmed ID were the evidence was found (as annotated in HPRD). In the field for the evidence type, vv stands for *in vivo*, vt for *in vitro* and 2H for yeast-two-hybrid assays.

| **Ensembl Id for SH2 containing protein** | **Ensembl ID for target protein** | **Experimental Evidence** | **Binding Site** | **Pubmed ID** |
| --- | --- | --- | --- | --- |
| ENSP00000274335 | ENSP00000222139 | vv |  | 7559499 |
| ENSP00000274335 | ENSP00000288032 | vt|2H|vv |  | 7982920 |
| ENSP00000274335 | ENSP00000324890 | vt|vv | Y191 | 8621607 |
| ENSP00000274335 | ENSP00000230882 | vv |  | 9632636 |
| ENSP00000274335 | ENSP00000294423 | vv |  | 9774657 |
| ENSP00000274335 | ENSP00000338934 | vt|vv | Y354 | 10377409 |
| ENSP00000274335 | ENSP00000264818 | vt|vv |  | 10995743 |
| ENSP00000274335 | ENSP00000346578 | vt|2H|vv | Y1039, Y1102, Y1108 | 10521483 |
| ENSP00000274335 | ENSP00000262908 | vt|vv | Y1117 | 11865050 |
| ENSP00000274335 | ENSP00000344798 | vt|vv | Y1062 | 10652352 |
| ENSP00000274335 | ENSP00000342681 | vv | Y487 | 9079809 |
| ENSP00000274335 | ENSP00000290277 | vv |  | 11812000 |
| ENSP00000274335 | ENSP00000303939 | vt|vv | Y201 | 7807015 |
| ENSP00000326032 | ENSP00000354566 | vt|vv | Y188, Y199 | 7761456 |
| ENSP00000326032 | ENSP00000278888 | vt|vv |  | 8071371 |
| ENSP00000326032 | ENSP00000222139 | vt|vv |  | 9852052 |
| ENSP00000326032 | ENSP00000221972 | vt|vv |  | 7500027 |
| ENSP00000326032 | ENSP00000085219 | vt |  | 8627166 |
| ENSP00000326032 | ENSP00000220597 | vt |  | 10790433 |
| ENSP00000354394 | ENSP00000288135 | vt|vv |  | 9355737 |
| ENSP00000354394 | ENSP00000275493 | vt|vv | Y978 | 12070153 |
| ENSP00000354394 | ENSP00000354394 | vt | Y701 | 9630226 |
| ENSP00000328213 | ENSP00000301178 | vv | Y821 | 9178760 |
| ENSP00000328213 | ENSP00000338171 | vt |  | 9195899 |
| ENSP00000328213 | ENSP00000220597 | vt |  | 10790433 |
| ENSP00000328213 | ENSP00000274335 | vv |  | 7504174 |
| ENSP00000328213 | ENSP00000263798 | 2H|vv |  | 10627473 |
| ENSP00000328213 | ENSP00000342681 | vt|vv |  | 7513045 |
| ENSP00000328213 | ENSP00000271610 | vt|vv |  | 8576115 |
| ENSP00000328213 | ENSP00000292588 | vt|vv |  | 8650207 |
| ENSP00000328213 | ENSP00000265010 | vt |  | 8879209 |
| ENSP00000328213 | ENSP00000313829 | vv |  | 9045636 |
| ENSP00000328213 | ENSP00000332816 | vt|vv |  | 9091579 |
| ENSP00000328213 | ENSP00000228307 | vv |  | 9488700 |
| ENSP00000328213 | ENSP00000264972 | vv | Y319 | 10318843 |
| ENSP00000328213 | ENSP00000305426 | vt|vv |  | 10455176 |
| ENSP00000328213 | ENSP00000226279 | vt|vv |  | 10636863 |
| ENSP00000328213 | ENSP00000276420 | vt|vv |  | 10799545 |
| ENSP00000328213 | ENSP00000345492 | vv |  | 10799879 |
| ENSP00000328213 | ENSP00000326032 | vv |  | 7539035 |
| ENSP00000353950 | ENSP00000264033 | vv |  | 8635998 |
| ENSP00000353950 | ENSP00000251849 | vt |  | 7517401 |
| ENSP00000353950 | ENSP00000332816 | vt|vv | Y402 | 8849729 |
| ENSP00000353950 | ENSP00000351245 | vt | Y94, Y451, Y453 | 9655255 |
| ENSP00000353950 | ENSP00000317272 | vv |  | 9837958 |
| ENSP00000353950 | ENSP00000305426 | vt|vv |  | 10455176 |
| ENSP00000353950 | ENSP00000330608 | vt|vv |  | 11483589 |
| ENSP00000353950 | ENSP00000301178 | vv | Y821 | 9178760 |
| ENSP00000353950 | ENSP00000344798 | vv |  | 10070972 |
| ENSP00000353950 | ENSP00000306124 | vt |  | 11834516 |
| ENSP00000353950 | ENSP00000338171 | vt |  | 9195899 |
| ENSP00000339007 | ENSP00000275493 | vt|vv |  | 1322798 |
| ENSP00000339007 | ENSP00000212292 | vv |  | 7500025 |
| ENSP00000339007 | ENSP00000346032 | vt |  | 7510700 |
| ENSP00000339007 | ENSP00000330608 | vv |  | 7664271 |
| ENSP00000339007 | ENSP00000261799 | vt|vv | Y716 | 7935391 |
| ENSP00000339007 | ENSP00000261937 | vt|vv |  | 7970715 |
| ENSP00000339007 | ENSP00000341189 | vt|vv | Y925 | 7997267 |
| ENSP00000339007 | ENSP00000305426 | vt |  | 10455176 |
| ENSP00000339007 | ENSP00000304895 | vv |  | 7488107 |
| ENSP00000339007 | ENSP00000326032 | vt|vv |  | 11964172 |
| ENSP00000339007 | ENSP00000353950 | vt|vv |  | 11964172 |
| ENSP00000339007 | ENSP00000258385 | vt|vv |  | 9668219 |
| ENSP00000339007 | ENSP00000085219 | vt|vv | Y807 | 11551923 |
| ENSP00000339007 | ENSP00000298467 | vt |  | 9516488 |
| ENSP00000339007 | ENSP00000296474 | vv | Y1360 | 8918464, 7488076 |
| ENSP00000339007 | ENSP00000355406 | vv |  | 9824671 |
| ENSP00000339007 | ENSP00000302452 | vt |  | 11882361 |
| ENSP00000339007 | ENSP00000220597 | vt |  | 10790433 |
| ENSP00000339007 | ENSP00000340944 | vv | Y542 | 8041791 |
| ENSP00000339007 | ENSP00000303507 | vt|vv | Y177 | 8112292 |
| ENSP00000339007 | ENSP00000313829 | vt|vv |  | 8576157 |
| ENSP00000339007 | ENSP00000324890 | vt|vv |  | 8576157 |
| ENSP00000339007 | ENSP00000345492 | vv |  | 8649391 |
| ENSP00000339007 | ENSP00000317272 | vt|vv | Y1374 | 8662889 |
| ENSP00000339007 | ENSP00000264731 | vt |  | 8695800 |
| ENSP00000339007 | ENSP00000244007 | vt|vv |  | 9281317 |
| ENSP00000339007 | ENSP00000286301 | vt|2H|vv | Y699, Y923 | 9380408 |
| ENSP00000339007 | ENSP00000288135 | vt|vv | Y703 | 10377264 |
| ENSP00000339007 | ENSP00000216373 | vv |  | 10940929 |
| ENSP00000339007 | ENSP00000351285 | vt|vv |  | 11964172 |
| ENSP00000339007 | ENSP00000254667 | vt|vv |  | 7518772, 10490839 |
| ENSP00000339007 | ENSP00000299293 | vt|vv | Y196, Y306, Y349, Y392 | 8780727, 12402043, 9182757 |
| ENSP00000339007 | ENSP00000327688 | vt |  | 8798570 |
| ENSP00000339007 | ENSP00000336919 | vt|vv |  | 11707405 |
