# Supplementary material for: Genome-Wide Prediction of SH2 Domain Targets Using Structural Information and the FoldX Algorithm
Source: PLoS Comput Biol. 2008 Apr 4;4(4):e1000052. doi: 10.1371/journal.pcbi.1000052 (PMC2271153; doi:10.1371/journal.pcbi.1000052)
Supplement: Table S6 — High-confidence predictions of SH2-mediated protein-protein interactions. (0.27 MB DOC) [file pcbi.1000052.s007.doc]

**Table S6:** **High-confidence predictions of SH2-mediated protein-protein interactions**. For 8 human SH2 domains with available complex structure we used Fold-X to predict the 10 most likely targets in the human proteome. We considered only tyrosines that have been predicted or shown experimentally to be phosphorylated (see methods). To further characterize the predicted interactions we compiled information on correlated expression, shared gene ontology terms, shared interaction partners and conservations of physical and genetic interactions. We used a naïve bayes approach to weight each evidence type in order to calculate the odds that a protein pair interacts *in vivo*, given this new information (see Methods). A posterior odd greater than 1 is equal to a probability higher than 50% that the interaction is a true *in vivo* interaction. For these interactions we could not find any conserved physical or genetic interactions. We found 27 interactions that were already known and documented in the Human Protein Reference Database (last checked on December 2007).

| SwissProt Id for SH2 containing protein | SwissProt Id for predicted Target | Ensembl Id for SH2 containing protein | Ensembl Id for predicted Target | Posterior Odds | Combined Likelihood ratio | GO LR | Conserved Int LR | Shared Int. LR | Correlated expression LR | Number of shared GO biological functions | Number of common interacting partners | Correlated expressed pair | Known int. |
| --- | --- | --- | --- | --- | --- | --- | --- | --- | --- | --- | --- | --- | --- |
| SRC_HUMAN | Q6S5L8_HUMAN | ENSP00000353950 | ENSP00000329668 | 0.0011 | 1 | 1 | 1 | 1 | 1 | 0 | 0 |  |  |
| SRC_HUMAN | B3AT_HUMAN | ENSP00000353950 | ENSP00000262418 | 0.0011 | 1 | 1 | 1 | 1 | 1 | 0 | 0 |  |  |
| SRC_HUMAN | TBA6_HUMAN | ENSP00000353950 | ENSP00000301072 | 2.23817 | 2034.7 | 1 | 1 | 2034.7 | 1 | 0 | >1 |  |  |
| SRC_HUMAN | FAK2_HUMAN | ENSP00000353950 | ENSP00000332816 | 254.8521 | 231683.8 | 13.2 | 1 | 17551.8 | 1 | GO_BP 1 | >7 |  | Yes |
| SRC_HUMAN | VIGLN_HUMAN | ENSP00000353950 | ENSP00000312042 | 0.0011 | 1 | 1 | 1 | 1 | 1 | 0 | 0 |  |  |
| SRC_HUMAN | FAK1_HUMAN | ENSP00000353950 | ENSP00000341189 | 254.8521 | 231683.8 | 13.2 | 1 | 17551.8 | 1 | GO_BP 1 | >7 |  | Yes |
| SRC_HUMAN | DEFI6_HUMAN | ENSP00000353950 | ENSP00000319831 | 0.0011 | 1 | 1 | 1 | 1 | 1 | 0 | 0 |  |  |
| SRC_HUMAN | IL3RB_HUMAN | ENSP00000353950 | ENSP00000262825 | 0.0011 | 1 | 1 | 1 | 1 | 1 | 0 | 0 |  |  |
| SRC_HUMAN | FABPH_HUMAN | ENSP00000353950 | ENSP00000311616 | 0.0011 | 1 | 1 | 1 | 1 | 1 | 0 | 0 |  |  |
| SRC_HUMAN | KPCD1_HUMAN | ENSP00000353950 | ENSP00000333568 | 7.567824 | 6879.84 | 13.2 | 1 | 521.2 | 1 | GO_BP 1 | >0 |  | Yes |
| LCK_HUMAN | ACHA6_HUMAN | ENSP00000328213 | ENSP00000276410 | 0.0011 | 1 | 1 | 1 | 1 | 1 | 0 | 0 |  |  |
| LCK_HUMAN | GAB1_HUMAN | ENSP00000328213 | ENSP00000262995 | 3.87013 | 3518.3 | 1 | 1 | 3518.3 | 1 | 0 | >2 |  |  |
| LCK_HUMAN | GNAS3_HUMAN | ENSP00000328213 | ENSP00000302237 | 0.0011 | 1 | 1 | 1 | 1 | 1 | 0 | 0 |  |  |
| LCK_HUMAN | Q86XU3_HUMAN | ENSP00000328213 | ENSP00000334051 | 0.0011 | 1 | 1 | 1 | 1 | 1 | 0 | 0 |  |  |
| LCK_HUMAN | ERBB2_HUMAN | ENSP00000328213 | ENSP00000269571 | 3.87013 | 3518.3 | 1 | 1 | 3518.3 | 1 | 0 | >2 |  |  |
| LCK_HUMAN | IRS1_HUMAN | ENSP00000328213 | ENSP00000304895 | 7.7077 | 7007 | 1 | 1 | 7007 | 1 | 0 | >4 |  |  |
| LCK_HUMAN | CD2L7_HUMAN | ENSP00000328213 | ENSP00000300647 | 0.0011 | 1 | 1 | 1 | 1 | 1 | 0 | 0 |  |  |
| LCK_HUMAN | M4K1_HUMAN | ENSP00000328213 | ENSP00000221409 | 0.00528 | 4.8 | 1 | 1 | 1 | 4.8 | 0 | 0 | co-exp. |  |
| LCK_HUMAN | Q8N556_HUMAN | ENSP00000328213 | ENSP00000351245 | 0.0011 | 1 | 1 | 1 | 1 | 1 | 0 | 0 |  |  |
| LCK_HUMAN | CSK21_HUMAN | ENSP00000328213 | ENSP00000217244 | 0.0011 | 1 | 1 | 1 | 1 | 1 | 0 | 0 |  |  |
| P85A_HUMAN | B3AT_HUMAN | ENSP00000274335 | ENSP00000262418 | 0.0011 | 1 | 1 | 1 | 1 | 1 | 0 | 0 |  |  |
| P85A_HUMAN | IRS1_HUMAN | ENSP00000274335 | ENSP00000304895 | 9.60344 | 8730.4 | 1 | 1 | 8730.4 | 1 | 0 | >5 |  | Yes |
| P85A_HUMAN | FGFR3_HUMAN | ENSP00000274335 | ENSP00000339824 | 2.23817 | 2034.7 | 1 | 1 | 2034.7 | 1 | 0 | >1 |  |  |
| P85A_HUMAN | KIT_HUMAN | ENSP00000274335 | ENSP00000288135 | 170.2731 | 154793.8 | 13.2 | 1 | 11726.8 | 1 | GO_BP 1 | >6 |  | Yes |
| P85A_HUMAN | EMAL4_HUMAN | ENSP00000274335 | ENSP00000320663 | 0.0011 | 1 | 1 | 1 | 1 | 1 | 0 | 0 |  |  |
| P85A_HUMAN | ERBB3_HUMAN | ENSP00000274335 | ENSP00000267101 | 51.08572 | 46441.56 | 13.2 | 1 | 3518.3 | 1 | GO_BP 1 | >2 |  | Yes |
| P85A_HUMAN | IKBA_HUMAN | ENSP00000274335 | ENSP00000216797 | 0.57332 | 521.2 | 1 | 1 | 521.2 | 1 | 0 | >0 |  | Yes |
| P85A_HUMAN | FGFR2_HUMAN | ENSP00000274335 | ENSP00000350166 | 0.0011 | 1 | 1 | 1 | 1 | 1 | 0 | 0 |  |  |
| P85A_HUMAN | FGFR1_HUMAN | ENSP00000274335 | ENSP00000311337 | 3.87013 | 3518.3 | 1 | 1 | 3518.3 | 1 | 0 | >2 |  | Yes |
| P85A_HUMAN | UFO_HUMAN | ENSP00000274335 | ENSP00000301178 | 51.08572 | 46441.56 | 13.2 | 1 | 3518.3 | 1 | GO_BP 1 | >2 |  | Yes |
| KSYK_HUMAN  (N-term) | B3AT_HUMAN | ENSP00000326032 | ENSP00000262418 | 0.0011 | 1 | 1 | 1 | 1 | 1 | 0 | 0 |  | Yes |
| KSYK_HUMAN  (N-term) | ERBB3_HUMAN | ENSP00000326032 | ENSP00000267101 | 7.567824 | 6879.84 | 13.2 | 1 | 521.2 | 1 | GO_BP 1 | >0 |  |  |
| KSYK_HUMAN  (N-term) | VAV_HUMAN | ENSP00000326032 | ENSP00000302269 | 254.8521 | 231683.8 | 13.2 | 1 | 17551.8 | 1 | GO_BP 1 | >7 |  | Yes |
| KSYK_HUMAN  (N-term) | CD19_HUMAN | ENSP00000326032 | ENSP00000313419 | 9.60344 | 8730.4 | 1 | 1 | 8730.4 | 1 | 0 | >5 |  | Yes |
| KSYK_HUMAN  (N-term) | CSF3R_HUMAN | ENSP00000326032 | ENSP00000355406 | 51.08572 | 46441.56 | 13.2 | 1 | 3518.3 | 1 | GO_BP 1 | >2 |  | Yes |
| KSYK_HUMAN  (N-term) | IKBA_HUMAN | ENSP00000326032 | ENSP00000216797 | 0.0011 | 1 | 1 | 1 | 1 | 1 | 0 | 0 |  |  |
| KSYK_HUMAN  (N-term) | AIP_HUMAN | ENSP00000326032 | ENSP00000279146 | 0.0011 | 1 | 1 | 1 | 1 | 1 | 0 | 0 |  |  |
| KSYK_HUMAN  (N-term) | ABL1_HUMAN | ENSP00000326032 | ENSP00000298467 | 1091.911 | 992646.5 | 113.7 | 1 | 8730.4 | 1 | GO_BP >1 | >5 |  |  |
| KSYK_HUMAN  (N-term) | ABL2_HUMAN | ENSP00000326032 | ENSP00000339209 | 0.0011 | 1 | 1 | 1 | 1 | 1 | 0 | 0 |  |  |
| KSYK_HUMAN  (N-term) | INSL3_HUMAN | ENSP00000326032 | ENSP00000222246 | 876.3655 | 796695.9 | 113.7 | 1 | 7007 | 1 | GO_BP >1 | >4 |  |  |
| KSYK_HUMAN  (C-term) | B3AT_HUMAN | ENSP00000326032 | ENSP00000262418 | 0.0011 | 1 | 1 | 1 | 1 | 1 | 0 | 0 |  | Yes |
| KSYK_HUMAN  (C-term) | ERBB3_HUMAN | ENSP00000326032 | ENSP00000267101 | 7.567824 | 6879.84 | 13.2 | 1 | 521.2 | 1 | GO_BP 1 | >0 |  |  |
| KSYK_HUMAN  (C-term) | VAV_HUMAN | ENSP00000326032 | ENSP00000302269 | 254.8521 | 231683.8 | 13.2 | 1 | 17551.8 | 1 | GO_BP 1 | >7 |  | Yes |
| KSYK_HUMAN  (C-term) | GELS_HUMAN | ENSP00000326032 | ENSP00000277268 | 2.23817 | 2034.7 | 1 | 1 | 2034.7 | 1 | 0 | >1 |  |  |
| KSYK_HUMAN  (C-term) | CD19_HUMAN | ENSP00000326032 | ENSP00000313419 | 9.60344 | 8730.4 | 1 | 1 | 8730.4 | 1 | 0 | >5 |  | Yes |
| KSYK_HUMAN  (C-term) | CD3E_HUMAN | ENSP00000326032 | ENSP00000354566 | 314.1794 | 285617.6 | 548 | 1 | 521.2 | 1 | GO_BP >2 | >0 |  | Yes |
| KSYK_HUMAN  (C-term) | JAK2_HUMAN | ENSP00000326032 | ENSP00000212292 | 2195.204 | 1995640 | 113.7 | 1 | 17551.8 | 1 | GO_BP >1 | >7 |  |  |
| KSYK_HUMAN  (C-term) | VAV2_HUMAN | ENSP00000326032 | ENSP00000317258 | 73.00946 | 66372.24 | 13.2 | 1 | 5028.2 | 1 | GO_BP 1 | >3 |  | Yes |
| KSYK_HUMAN  (C-term) | FABPH_HUMAN | ENSP00000326032 | ENSP00000311616 | 0.0011 | 1 | 1 | 1 | 1 | 1 | 0 | 0 |  |  |
| KSYK_HUMAN  (C-term) | IKBA_HUMAN | ENSP00000326032 | ENSP00000216797 | 0.0011 | 1 | 1 | 1 | 1 | 1 | 0 | 0 |  |  |
| STAT1_HUMAN | FGFR2_HUMAN | ENSP00000354394 | ENSP00000350166 | 0.0011 | 1 | 1 | 1 | 1 | 1 | 0 | 0 |  |  |
| STAT1_HUMAN | FGFR1_HUMAN | ENSP00000354394 | ENSP00000311337 | 0.0011 | 1 | 1 | 1 | 1 | 1 | 0 | 0 |  |  |
| STAT1_HUMAN | O14654_HUMAN | ENSP00000354394 | ENSP00000247066 | 0.00528 | 4.8 | 1 | 1 | 1 | 4.8 | 0 | 0 | co-exp. |  |
| STAT1_HUMAN | CHD1_HUMAN | ENSP00000354394 | ENSP00000284049 | 0.0011 | 1 | 1 | 1 | 1 | 1 | 0 | 0 |  |  |
| STAT1_HUMAN | CHD2_HUMAN | ENSP00000354394 | ENSP00000311700 | 0.0011 | 1 | 1 | 1 | 1 | 1 | 0 | 0 |  |  |
| STAT1_HUMAN | DUS3_HUMAN | ENSP00000354394 | ENSP00000226004 | 0.0011 | 1 | 1 | 1 | 1 | 1 | 0 | 0 |  | Yes |
| STAT1_HUMAN | GAB2_HUMAN | ENSP00000354394 | ENSP00000302452 | 0.57332 | 521.2 | 1 | 1 | 521.2 | 1 | 0 | >0 |  |  |
| STAT1_HUMAN | DOK1_HUMAN | ENSP00000354394 | ENSP00000233668 | 0.57332 | 521.2 | 1 | 1 | 521.2 | 1 | 0 | >0 |  |  |
| STAT1_HUMAN | EMD_HUMAN | ENSP00000354394 | ENSP00000218240 | 0.0011 | 1 | 1 | 1 | 1 | 1 | 0 | 0 |  |  |
| STAT1_HUMAN | LCK_HUMAN | ENSP00000354394 | ENSP00000328213 | 18.57662 | 16887.84 | 1 | 1 | 3518.3 | 4.8 | 0 | >2 | co-exp. | Yes |
| SH21A_HUMAN | WASL_HUMAN | ENSP00000331181 | ENSP00000223023 | 0.0011 | 1 | 1 | 1 | 1 | 1 | 0 | 0 |  |  |
| SH21A_HUMAN | RS10_HUMAN | ENSP00000331181 | ENSP00000346060 | 0.0011 | 1 | 1 | 1 | 1 | 1 | 0 | 0 |  |  |
| SH21A_HUMAN | SLAF1_HUMAN | ENSP00000331181 | ENSP00000342054 | 0.00528 | 4.8 | 1 | 1 | 1 | 4.8 | 0 | 0 | co-exp. | Yes |
| SH21A_HUMAN | FAK1_HUMAN | ENSP00000331181 | ENSP00000341189 | 0.0011 | 1 | 1 | 1 | 1 | 1 | 0 | 0 |  |  |
| SH21A_HUMAN | ITPR1_HUMAN | ENSP00000331181 | ENSP00000349597 | 0.0011 | 1 | 1 | 1 | 1 | 1 | 0 | 0 |  |  |
| SH21A_HUMAN | TRI25_HUMAN | ENSP00000331181 | ENSP00000323889 | 0.0011 | 1 | 1 | 1 | 1 | 1 | 0 | 0 |  |  |
| SH21A_HUMAN | CHSS3_HUMAN | ENSP00000331181 | ENSP00000302629 | 0.0011 | 1 | 1 | 1 | 1 | 1 | 0 | 0 |  |  |
| SH21A_HUMAN | DDX5_HUMAN | ENSP00000331181 | ENSP00000225792 | 0.0011 | 1 | 1 | 1 | 1 | 1 | 0 | 0 |  |  |
| SH21A_HUMAN | 5HT6R_HUMAN | ENSP00000331181 | ENSP00000289753 | 0.0011 | 1 | 1 | 1 | 1 | 1 | 0 | 0 |  |  |
| SH21A_HUMAN | ABLM1_HUMAN | ENSP00000331181 | ENSP00000338190 | 0.0011 | 1 | 1 | 1 | 1 | 1 | 0 | 0 |  |  |
| NCK1_HUMAN | FLT3_HUMAN | ENSP00000288986 | ENSP00000241453 | 0.0011 | 1 | 1 | 1 | 1 | 1 | 0 | 0 |  | Yes |
| NCK1_HUMAN | CSK21_HUMAN | ENSP00000288986 | ENSP00000217244 | 0.57332 | 521.2 | 1 | 1 | 521.2 | 1 | 0 | >0 |  |  |
| NCK1_HUMAN | Q5VZS7_HUMAN | ENSP00000288986 | ENSP00000343926 | 0.0011 | 1 | 1 | 1 | 1 | 1 | 0 | 0 |  |  |
| NCK1_HUMAN | GLGB_HUMAN | ENSP00000288986 | ENSP00000264326 | 0.00528 | 4.8 | 1 | 1 | 1 | 4.8 | 0 | 0 | co-exp. |  |
| NCK1_HUMAN | VIGLN_HUMAN | ENSP00000288986 | ENSP00000312042 | 0.0011 | 1 | 1 | 1 | 1 | 1 | 0 | 0 |  |  |
| NCK1_HUMAN | BCAR1_HUMAN | ENSP00000288986 | ENSP00000162330 | 3.87013 | 3518.3 | 1 | 1 | 3518.3 | 1 | 0 | >2 |  | Yes |
| NCK1_HUMAN | HNRPQ_HUMAN | ENSP00000288986 | ENSP00000257767 | 0.0011 | 1 | 1 | 1 | 1 | 1 | 0 | 0 |  |  |
| NCK1_HUMAN | ABL1_HUMAN | ENSP00000288986 | ENSP00000298467 | 126.7654 | 115241.3 | 13.2 | 1 | 8730.4 | 1 | GO_BP 1 | >5 |  | Yes |
| NCK1_HUMAN | KIT_HUMAN | ENSP00000288986 | ENSP00000288135 | 0.57332 | 521.2 | 1 | 1 | 521.2 | 1 | 0 | >0 |  |  |
| NCK1_HUMAN | RBM46_HUMAN | ENSP00000288986 | ENSP00000281722 | 0.0011 | 1 | 1 | 1 | 1 | 1 | 0 | 0 |  |  |
| GRB2_HUMAN | CD28_HUMAN | ENSP00000339007 | ENSP00000324890 | 5.53102 | 5028.2 | 1 | 1 | 5028.2 | 1 | 0 | >3 |  | Yes |
| GRB2_HUMAN | IRS1_HUMAN | ENSP00000339007 | ENSP00000304895 | 19.30698 | 17551.8 | 1 | 1 | 17551.8 | 1 | 0 | >7 |  | Yes |
| GRB2_HUMAN | IRS2_HUMAN | ENSP00000339007 | ENSP00000329289 | 9.60344 | 8730.4 | 1 | 1 | 8730.4 | 1 | 0 | >5 |  | Yes |
| GRB2_HUMAN | GNAI2_HUMAN | ENSP00000339007 | ENSP00000312999 | 0.57332 | 521.2 | 1 | 1 | 521.2 | 1 | 0 | >0 |  |  |
| GRB2_HUMAN | HGS_HUMAN | ENSP00000339007 | ENSP00000331201 | 2.23817 | 2034.7 | 1 | 1 | 2034.7 | 1 | 0 | >1 |  |  |
| GRB2_HUMAN | RGS1_HUMAN | ENSP00000339007 | ENSP00000204113 | 0.0011 | 1 | 1 | 1 | 1 | 1 | 0 | 0 |  |  |
| GRB2_HUMAN | EPHA8_HUMAN | ENSP00000339007 | ENSP00000166244 | 0.0011 | 1 | 1 | 1 | 1 | 1 | 0 | 0 |  |  |
| GRB2_HUMAN | RON_HUMAN | ENSP00000339007 | ENSP00000296474 | 5.53102 | 5028.2 | 1 | 1 | 5028.2 | 1 | 0 | >3 |  | Yes |
| GRB2_HUMAN | UFO_HUMAN | ENSP00000339007 | ENSP00000301178 | 2.23817 | 2034.7 | 1 | 1 | 2034.7 | 1 | 0 | >1 |  | Yes |
| GRB2_HUMAN | BCR_HUMAN | ENSP00000339007 | ENSP00000303507 | 126.7654 | 115241.3 | 13.2 | 1 | 8730.4 | 1 | GO_BP 1 | >5 |  | Yes |
