# Supplementary material for: Genome-Wide Prediction of SH2 Domain Targets Using Structural Information and the FoldX Algorithm
Source: PLoS Comput Biol. 2008 Apr 4;4(4):e1000052. doi: 10.1371/journal.pcbi.1000052 (PMC2271153; doi:10.1371/journal.pcbi.1000052)
Supplement: Text S1 — Supplementary methods. (0.18 MB DOC) [file pcbi.1000052.s008.doc]

**Supplementary Methods: Evidence integration using a naïve bayes approach.**

We have used a naïve bayes approach similar to the described by Rhodes and colleagues [1] to integrate the different available information into a predicted likelihood that a given protein pair interacts. We have extracted from the human protein reference database [2] protein pairs tagged as interacting *in vivo* that we considered as our Gold Positive Standard (GPS). The dataset downloaded on 27/02/2006 contains 8235 *in vivo* interactions among 3861 proteins. We considered that proteins annotated in the Gene Ontology database [3] to belong to the plasma membrane are less likely to interact to proteins belonging to the nucleus and we built a set of negative interactions (our Gold Negative Standard – GNS) from all protein pairs among these proteins (2663352 negative interactions).

**Interaction annotation across species**

We considered that protein interaction information is to some extent conserved across species [4,5]. We have compiled genetic and physical interactions for *S. cerevisiae*, *D. melanogaster* and *C. elegans* from BIND [6], BioGRID [7], Flybase [8] and Wormbase (http://www.wormbase.org). We have used BLAST [9] to determine protein homology between species. To make the most of this information we used different homology/orthology assignments determining for each the predictive power of identifying human in vivo interactions. We transferred the annotation across species considering four bins of increasing confidence: 1) the best blast hit from each of the species to H. sapiens; 2) the best blast hit from each of the species to *H. sapiens* with E value <1E-10; 3) indirect reciprocal best blast hit; 4) the reciprocal best blast hit. We defined a human protein as an indirect reciprocal best hit of a protein A in another species when the human protein’s best blast hit (A’) likely originated by duplication from A. When A’ is the best blast hit of A in that genome with an E value <1E-30.

**Human gene co-expression**

Gene expression information has been used to increase the confidence in predicting protein interactions [1]. Co-expressed human gene pairs were taken from the literature [10,11] and stratified into two bins according to the level of confidence of co-expression. The smaller bin of high confidence co-expressed genes was taken from [10] that can be found online (www.bcgsc.ca/project/bomge/coexpression/).

**Gene Ontology annotations**

Proteins pairs that participate in the same biological processes or have similar molecular functions are more likely to interact. We have binned protein pairs with increasing number of shared GO annotations [3] on biological processes or molecular functions and we tested the predictive power of each bin (see tables 3 and 4).

**Shared binding partners**

Protein pairs sharing binding partners are more probably interacting than random proteins given that they more likely belong to the same pathway or complex. We created a human interaction network combining the interactions in the Human Protein Reference Database [2] with two recent yeast-two-hybrid studies [12,13]. We then binned human protein pairs with increasing number of shared partners and for each bin calculated the likelihood of detecting an in vivo interaction.

**Naïve bayes approach**

We have used a similar bayes approach as the one used to predict yeast complexes [14] and human protein-protein interactions [1]. We want to determine how the evidence observed impacts on the odds that a pair of proteins interacts. Defining the prior odds (O_prior_) as the odds that two random proteins interact, the posterior odds (O_posterior_) are the odds that two proteins interact given new predictive evidences. The posterior odds can be calculated as:

O_posterior_ = O_prior_ * L(f1…fn)

Where L(f1…fn) is the likelihood ratio given by:

L = P(f1…fn | pos) / P(f1…fn | neg)

Where fi is the evidence found for a protein interaction in dataset i.

Assuming that the datasets are conditionally independent L can be calculated as the product of individual likelihood ratios:

LR = LRint_transfer × LR_co-exp_ × LR_GO_ × LRshared_int

Where LRint_transfer is the likelihood ratio obtained from interaction transfer, LR_co-exp_ is the likelihood ratio obtained from gene co-expression information, LR_GO_ is the likelihood ratio obtained from Gene Ontology information and LRshared_int is the likelihood ratio obtained from the number of shared binding partners. Within each of the evidence types we have different confidence bins for which we calculated likelihood ratios (see tables 1 to 5) by determining the overlap with the gold positive and negative sets. When multiple evidences were observed within one of the data types we have taken the highest likelihood value.

**Estimating prior odds**

We estimated the prior odds that two proteins interact *in vivo* using the interactions in the HPRD that we included in our Gold Positive Set. As we mentioned above, the dataset contains 8235 *in vivo* interactions among 3861 proteins. The odds that two of these proteins interact are approximately 0.0011 or approximately 1 in 900. Therefore the posterior odds that two proteins interact *in vivo* are:

O_posterior_ = 0.0011 * L(f1…fn)

In order to have 50% or higher likelihood of being a true interaction the likelihood ratio should equal or above 900. This should be considered a conservative estimate of the posterior odds for two different reasons. The dataset used is probably not complete and therefore the likelihood that two random proteins will interact is possibly higher. Also, we are in this study focusing on the interactions of Ras containing proteins with Rbd containing proteins that are more likely to interact than any random proteins. There are 10 interactions in our GPS between one of the 20 Ras containing proteins and one of the 52 Rbd containing proteins. The odds that these proteins interact without any other evidence is approximately 0.0096, about 9 times higher than for a random protein pair. We have not used this value as prior odds because it might not be representative of the true value given the small number of proteins and interactions currently studied.

**Tables of likelihood ratios**

**Estimation of Likelihood Ratio for interactions transfer across species**.

We have calculated the overlap of predicted human interactions with the GPS and GNS and used this information to calculate a likelihood ratio for this evidence type. The interactions were predicted using available physical and genetic interactions for *S. cerevisiae*, *D. melanogaster* and *C. elegans*. We have used four types of homology/orthology detection using BLAST: RBH – reciprocal best blast hit; IRBH – indirect reciprocal best blast hit; best homolog with an E value smaller than 1E-10 and simple the best blast hit with no E value cut off. We defined a human protein as an indirect reciprocal best hit of a protein A in another species when the human protein’s best blast hit (A’) likely originated by duplication from A. When A’ is the best blast hit of A in that genome with an E value <1E-30.

| Interaction type | Homology type | N(pos) | N(neg) | p(pos) | p(neg) | LR p(pos)/p(neg) |
| --- | --- | --- | --- | --- | --- | --- |
| *D.melanogaster* Physical interactions | RBH | 59 | 38 | 0.007165 | 1.43E-05 | 502.1499 |
|  | IRBH | 52 | 52 | 0.006315 | 1.95E-05 | 323.4186 |
|  | best homolog <1E-10 | 53 | 73 | 0.006436 | 2.74E-05 | 234.8107 |
|  | best blast hit | 57 | 166 | 0.006922 | 6.23E-05 | 111.0534 |
| *D.melanogaster* Genetic interactions | RBH | 79 | 74 | 0.009593 | 2.78E-05 | 345.2712 |
|  | IRBH | 83 | 74 | 0.010079 | 2.78E-05 | 362.7533 |
|  | best homolog <1E-10 | 89 | 82 | 0.010808 | 3.08E-05 | 351.0275 |
|  | best blast hit | 90 | 101 | 0.010929 | 3.79E-05 | 288.1948 |
| *S. cerevisiae* Physical interactions | RBH | 121 | 10 | 0.014693 | 3.75E-06 | 3913.365 |
|  | IRBH | 133 | 16 | 0.016151 | 6.01E-06 | 2688.417 |
|  | best homolog <1E-10 | 150 | 33 | 0.018215 | 1.24E-05 | 1470.084 |
|  | best blast hit | 181 | 111 | 0.021979 | 4.17E-05 | 527.3762 |
| *S. cerevisiae* Complexes | RBH | 213 | 38 | 0.025865 | 1.43E-05 | 1812.846 |
|  | IRBH | 233 | 43 | 0.028294 | 1.61E-05 | 1752.477 |
|  | best homolog <1E-10 | 255 | 79 | 0.030965 | 2.97E-05 | 1043.946 |
|  | best blast hit | 310 | 165 | 0.037644 | 6.2E-05 | 607.6349 |
| *S. cerevisiae* Genetic interactions | RBH | 93 | 11 | 0.011293 | 4.13E-06 | 2734.357 |
|  | IRBH | 104 | 76 | 0.012629 | 2.85E-05 | 442.5728 |
|  | best homolog <1E-10 | 109 | 82 | 0.013236 | 3.08E-05 | 429.9101 |
|  | best blast hit | 141 | 182 | 0.017122 | 6.83E-05 | 250.5605 |
| *C. elegans* Physical interactions | RBH | 18 | 9 | 0.002186 | 3.38E-06 | 646.8372 |
|  | IRBH | 18 | 12 | 0.002186 | 4.51E-06 | 485.1279 |
|  | best homolog <1E-10 | 19 | 20 | 0.002307 | 7.51E-06 | 307.2477 |
|  | best blast hit | 20 | 57 | 0.002429 | 2.14E-05 | 113.4802 |
| *C. elegans* Genetic interactions | RBH | 73 | 37 | 0.008865 | 1.39E-05 | 638.0961 |
|  | IRBH | 80 | 38 | 0.009715 | 1.43E-05 | 680.8812 |
|  | best homolog <1E-10 | 91 | 47 | 0.01105 | 1.76E-05 | 626.1934 |
|  | best blast hit | 99 | 55 | 0.012022 | 2.07E-05 | 582.1534 |

**Estimation of Likelihood Ratio for protein pairs sharing interaction partners**. We binned human protein pairs with increasing number of shared interactions and for each bin calculated the overlap with the GPS and GNS and used this information to calculate a likelihood ratio.

| Minimal N of shared Interactions | N(pos) | N(neg) | p(pos) | p(neg) | LR=p(pos)/p(neg) |
| --- | --- | --- | --- | --- | --- |
| 1 | 4114 | 5445 | 1.065527 | 0.002044 | 521.1889 |
| 2 | 2286 | 775 | 0.592075 | 0.000291 | 2034.714 |
| 3 | 1372 | 269 | 0.355348 | 0.000101 | 3518.282 |
| 4 | 882 | 121 | 0.228438 | 4.54E-05 | 5028.193 |
| 5 | 579 | 57 | 0.149961 | 2.14E-05 | 7007.006 |
| 6 | 405 | 32 | 0.104895 | 1.2E-05 | 8730.393 |
| 7 | 289 | 17 | 0.074851 | 6.38E-06 | 11726.75 |
| 8 | 229 | 9 | 0.059311 | 3.38E-06 | 17551.8 |

**Estimation of Likelihood Ratio for protein pairs sharing biological processes**. We binned human protein pairs with increasing number of shared biological processes as determined by GO annotations and for each bin calculated the overlap with the GPS and GNS and used this information to calculate a likelihood ratio.

| Minimal number of shared GO process | N(pos) | N(neg) | p(pos) | p(neg) | LR=p(pos)/p(neg) |
| --- | --- | --- | --- | --- | --- |
| 1 | 3032 | 74242 | 0.368185 | 0.027875 | 13.20823 |
| 2 | 1141 | 3244 | 0.138555 | 0.001218 | 113.7548 |
| 3 | 361 | 213 | 0.043837 | 8E-05 | 548.1413 |
| 4 | 100 | 18 | 0.012143 | 6.76E-06 | 1796.77 |
| 5 | 36 | 1 | 0.004372 | 3.75E-07 | 11643.07 |

**Estimation of Likelihood Ratio for protein pairs sharing molecular functions**. We binned human protein pairs with increasing number of shared molecular functions as determined by GO annotations and for each bin calculated the overlap with the GPS and GNS and used this information to calculate a likelihood ratio.

| Minimal number of shared GO functions | N(pos) | N(neg) | p(pos) | p(neg) | LR=p(pos)/p(neg) |
| --- | --- | --- | --- | --- | --- |
| 1 | 3945 | 321961 | 0.479053 | 0.120886 | 3.96286 |
| 2 | 1244 | 40577 | 0.151063 | 0.015235 | 9.91529 |
| 3 | 545 | 6131 | 0.066181 | 0.002302 | 28.74949 |
| 4 | 302 | 1641 | 0.036673 | 0.000616 | 59.52006 |
| 5 | 157 | 716 | 0.019065 | 0.000269 | 70.9172 |
| 6 | 61 | 145 | 0.007407 | 5.44E-05 | 136.0589 |
| 7 | 15 | 20 | 0.001821 | 7.51E-06 | 242.5639 |
| 8 | 6 | 4 | 0.000729 | 1.5E-06 | 485.1279 |

**Estimation of Likelihood Ratio for co-expressed protein pairs**. We binned human proteins pairs into co-expressed pairs or a set of high confidence co-expression and for both cases calculated the overlap with the GPS and GNS. We have used this information to calculate likelihood ratio scores.

| Co-expression | N(pos) | N(neg) | p(pos) | p(neg) | LR=p(pos)/p(neg) |
| --- | --- | --- | --- | --- | --- |
| Co-expressed genes | 825 | 55359 | 0.100182 | 0.020785 | 4.819818 |
| High-confidence co-expressed genes | 79 | 408 | 0.009593 | 0.000153 | 62.62272 |

**References**

1. Rhodes DR, Tomlins SA, Varambally S, Mahavisno V, Barrette T, et al. (2005) Probabilistic model of the human protein-protein interaction network. Nat Biotechnol 23: 951-959.

2. Peri S, Navarro JD, Amanchy R, Kristiansen TZ, Jonnalagadda CK, et al. (2003) Development of human protein reference database as an initial platform for approaching systems biology in humans. Genome Res 13: 2363-2371.

3. Ashburner M, Ball CA, Blake JA, Botstein D, Butler H, et al. (2000) Gene ontology: tool for the unification of biology. The Gene Ontology Consortium. Nat Genet 25: 25-29.

4. Walhout AJ, Sordella R, Lu X, Hartley JL, Temple GF, et al. (2000) Protein interaction mapping in C. elegans using proteins involved in vulval development. Science 287: 116-122.

5. Matthews LR, Vaglio P, Reboul J, Ge H, Davis BP, et al. (2001) Identification of potential interaction networks using sequence-based searches for conserved protein-protein interactions or "interologs". Genome Res 11: 2120-2126.

6. Alfarano C, Andrade CE, Anthony K, Bahroos N, Bajec M, et al. (2005) The Biomolecular Interaction Network Database and related tools 2005 update. Nucleic Acids Res 33: D418-424.

7. Stark C, Breitkreutz BJ, Reguly T, Boucher L, Breitkreutz A, et al. (2006) BioGRID: a general repository for interaction datasets. Nucleic Acids Res 34: D535-539.

8. Grumbling G, Strelets V (2006) FlyBase: anatomical data, images and queries. Nucleic Acids Res 34: D484-488.

9. Altschul SF, Gish W, Miller W, Myers EW, Lipman DJ (1990) Basic local alignment search tool. J Mol Biol 215: 403-410.

10. Lee HK, Hsu AK, Sajdak J, Qin J, Pavlidis P (2004) Coexpression analysis of human genes across many microarray data sets. Genome Res 14: 1085-1094.

11. Griffith OL, Pleasance ED, Fulton DL, Oveisi M, Ester M, et al. (2005) Assessment and integration of publicly available SAGE, cDNA microarray, and oligonucleotide microarray expression data for global coexpression analyses. Genomics 86: 476-488.

12. Rual JF, Venkatesan K, Hao T, Hirozane-Kishikawa T, Dricot A, et al. (2005) Towards a proteome-scale map of the human protein-protein interaction network. Nature 437: 1173-1178.

13. Stelzl U, Worm U, Lalowski M, Haenig C, Brembeck FH, et al. (2005) A human protein-protein interaction network: a resource for annotating the proteome. Cell 122: 957-968.

14. Jansen R, Yu H, Greenbaum D, Kluger Y, Krogan NJ, et al. (2003) A Bayesian networks approach for predicting protein-protein interactions from genomic data. Science 302: 449-453.
